# Distinctive phenotype for HLA-E- versus HLA-A2-restricted memory CD8 αβT cells in the course of HCMV infection discloses features shared with NKG2C^+^CD57^+^NK and δ2^-^γδT cell subsets

**DOI:** 10.3389/fimmu.2022.1063690

**Published:** 2022-12-01

**Authors:** Amélie Rousselière, Nathalie Gérard, Laurence Delbos, Pierrick Guérif, Magali Giral, Céline Bressollette-Bodin, Béatrice Charreau

**Affiliations:** ^1^ Nantes Université, CHU Nantes, Inserm, Centre de Recherche Translationnelle en Transplantation et Immunologie, UMR 1064, Nantes, France; ^2^ CHU Nantes, Nantes Université, Institut de Transplantation Urologie Néphrologie (ITUN), Nantes, France; ^3^ CHU Nantes, Nantes Université, Laboratoire de Virologie, Nantes, France

**Keywords:** HCMV, HLA-E, CD8 T cells, CD56, PD-1, NK, γδT

## Abstract

The human cytomegalovirus (HCMV) triggers both innate and adaptive immune responses, including protective CD8^+^ αβT cells (CD8T) that contributes to the control of the infection. In addition to CD8T restricted by classical HLA class Ia molecules, HCMV also triggers CD8T recognizing peptides from the HCMV UL40 leader peptide and restricted by HLA-E molecules (HLA-E_UL40_ CD8T). This study investigated the frequency, phenotype and functions of HLA-E_UL40_ CD8T in comparison to the immunodominant HLA-A2_pp65_ CD8T upon acute (primary or secondary infection) or chronic infection in kidney transplant recipients (KTR) and in seropositive (HCMV^+^) healthy volunteer (HV) hosts. The frequency of hosts with detected HLA-E_UL40_ CD8T was similar after a primary infection (24%) and during viral latency in HCMV+ HV (26%) and equal to the frequency of HLA-A2_pp65_ CD8T cells in both conditions (29%). Both CD8T subsets vary from 0.1% to >30% of total circulating CD8T according to the host. Both HLA-E_UL40_ and HLA-A2_pp65_ CD8T display a phenotype specific of CD8^+^ TEMRA (CD45RA^+^/CCR7^-^) but HLA-E_UL40_ CD8T express distinctive level for CD3, CD8 and CD45RA. Tim3, Lag-3, 4-1BB, and to a lesser extend 2B4 are hallmarks for T cell priming post-primary infection while KLRG1 and Tigit are markers for restimulated and long lived HCMV-specific CD8T responses. These cell markers are equally expressed on HLA-E_UL40_ and HLA-A2_pp65_ CD8T. In contrast, CD56 and PD-1 are cell markers discriminating memory HLA-E- from HLA-A2-restricted CD8T. Long lived HLA-E_UL40_ display higher proliferation rate compared to HLA-A2_pp65_ CD8T consistent with elevated CD57 expression. Finally, a comparative immunoprofiling indicated that HLA-E_UL40_ CD8T, divergent from HLA-A2_pp65_ CD8T, share the expression of CD56, CD57, NKG2C, CD158 and the lack of PD-1 with NKG2C^+^CD57+ NK and δ2^-^γδT cells induced in response to HCMV and thus defines a common immunopattern for these subsets.

## Introduction

Human cytomegalovirus (HCMV) is a ubiquitous virus found in > 50% of the world’s population (1), commonly characterized by latency and lifelong persistence after primary infection in the immunocompetent hosts (2). By using a large spectrum of immune escape strategies, HCMV can survive in the host despite robust innate and adaptive immune responses and cause chronic infection (3). Although more often asymptomatic in immunocompetent hosts, HCMV infection can cause life threatening infections in individuals with a suppressed immune system or immunodeficiency disorders. HCMV remains a challenging obstacle in transplant recipients due to immunosuppresssive regimen (2). HCMV trigger innate and adaptive immune responses.

CD8^+^ T cells play an essential role in the host immune response to HCMV and other viruses by recognizing and eliminating infected cells (4–6). Recognition is mediated by the αβT cell receptor (TCR), which binds viral peptides presented by major histocompatibility complex (MHC) class I molecules on infected cells (7). After infection, antigen-specific CD8^+^ αβT (CD8T) cells undergo clonal expansion and acquire effector functions to eliminate infected cells (6). The effectiveness of the T cell response to a given virus relies on highly diverse αβ TCR repertoires able to recognize multiple viral epitopes and assure protection from viral escape (8). T-cell responses against HCMV naturally focus not only on one, but multiple antigens. A total of 151 HCMV open reading frames (ORFs) out of the 213 HCMV ORFs, were found to be immunogenic for CD4^+^ and/or CD8T cells (9). The CD8T cell response to HCMV has been studied extensively (2, 4, 8). The tegument protein pp65 (UL83) accounts for 70%–90% of the CD8^+^ T cell response to HCMV (9–11). The dominant epitope in human leukocyte antigen (HLA)-A2^+^ individuals corresponds to AA 495–503 of pp65 (NLVPMVATV) (12). In addition to conventional anti-HCMV CD8T cell responses defined by peptide presentation by classical HLA class I molecules exemplified by HLA-A*02:01pp65 (referred to as HLA-A2_pp65_), unconventional CD8T cells responses that recognize signal peptides from HCMV UL40 leader peptides presented by HLA-E molecules (13, 14) (referred to as HLA-E_UL40_) have been also reported (15–19). However, studies have not been rigourously performed comparing the phenotype, protective abilities and functional activities of HLA-E_UL40_ versus conventional HLA-A2_pp65_ memory CD8T cells during the various phases of HCMV chronic infections.

We have previously shown that HLA-E_UL40_ CD8^+^T responses are frequently induced in response to HCMV infection in kidney transplant recipients (KTR) as well as in healthy subjects which may represent a large fraction of the CD8T cells compartiment post-infection that persist for life (19). The phenotype and functions of HLA-E_UL40_ CD8T cells still remain poorly described (20). The present study provides a refined phenotype characterization of HLA-E_UL40_ CD8T in comparison to HLA-A2_pp65_ CD8T cells responses during the acute and chronic phases of HCMV infection in KTR and healthy HCMV seropositive (HCMV^+^) subjects (healthy volunteers, HV). Our findings indicate that memory HLA-E_UL40_ CD8T cells responses occurs rapidly post-infection and may preceed the development of HLA-A2_pp65_ CD8T cells responses reflecting the rapid and earlier expression of UL40 compared to pp65 in HCMV infected cells. Here we show that circulating HLA-E_UL40_ CD8Tand HLA-A2_pp65_ CD8T cells are effector memory CD8T re-expressing CD45RA (TEMRA) which share common phenotype traits evolving according infection phase (primary infection, reactivation and chronic infection). We also found qualitive and quantitative changes between the two responses and we identified a set of markers that distingish HLA-E_UL40_ from HLA-A2_pp65_ CD8T cells responses which may be indicative of specific functions, regulations and cell targets. A comparative immunophenotyping further indicates that divergent from HLA-A2_pp65_ CD8T, HLA-E_UL40_ CD8T share with NKG2C^+^CD57^+^ NK and δ2^-^γδT cells the expression of CD56, CD57, NKG2C, CD158 and the lack of PD-1 which thus define a common immunopattern for these subsets induced in response to HCMV.

## Materials and methods

### Study approval and ethics

Banked biological samples (peripheral blood mononulear cells, PBMC) were issued from the DIVAT biocollection (CNIL agreement n°891735, French Health Minister Project n°02G55) and stored at the Centre de Ressources Biologiques (CRB, CHU Nantes, France). This retrospective study was performed according to the guidelines of the local and national ethics committees (CCPRB, CHU Nantes, France). Blood samples collected from anonymous healthy volunteers (HV, n=35) were obtained from the Etablissement Français de Sang (EFS Pays de la Loire, Nantes, France) and collected with donor’s specific and written informed consent for research use.

### Patients and samples

A total of 21 patients who underwent kidney transplantation in our institute (Institute for Transplantation Urology Nephrology, ITUN, CHU Nantes, France) between 2009 and 2017 were retrospectively enrolled in our study. The cohort includes HCMV seronegative (HCMV^-^) transplant recipients (R^-^) at the time of transplantation with a primary HCMV infection post-transplantation (n=17) and seropositive (HCMV^+^) transplant recipients (R^+^, n=4) with a HCMV reactivation post-transplantation. Characteristics of the patients are provided in **Table 1**. No statistical differences were found between HV and KTR patients related to age or gender ratio.

**Table 1:** Characteristics of the HCMV^+^ hosts.

	HCMV+			
	Primary-infection(n = 17)	Reinfection/Reactivation(n = 4)	Latent infection(n = 35)	p-value
***Transplant HCMV serologic status (M12 post-transplantation)***				
D+/R-	16/17	0	NA	
D-/R-	1/17	0	NA	
D+/R+	0	2/4	NA	
D-/R+	0	2/4	NA	
***Transplant DONORS***				
Age [years; median (Q1-Q3)]	49.4 (44-63)	58.75 (57-63)	NA	0.4222^1^
Gender [Male/Female; (% of Male)]	12/5 (70.6%)	2/2 (50%)	NA	0.5743^3^
Donor status [Deceased/Living (% of deceased donors)]	16/1 (94.1%)	4/0 (100%)	NA	>0.9999^3^
***Transplant RECIPIENTS***			***HEALTHY VOLUNTEERS***	
Age [years; median (Q1-Q3)]	48.4 (40.1-60.4)	60.3 (51-61.4)	51.2 (37-64)	0.3100^2^
Gender [Male/Female; (% of Male)]	12/5 (70.6%)	4/0 (100%)	19/16 (54.3%)	0.1436^4^
Transplant [Kidney/Pancreas-Kidney; (% of Kidney only)]	16/1 (94.1%)	4/0 (100%)	NA	>0,9999^3^
Cold ischemia [minutes; median (Q1-Q3)]	978.3 (643-1274)	1078.8 (868-974)	NA	0.6353^1^
Serum creatinine at M12 [µmol/L; median (Q1-Q3)]	79.1 (6.8-139)	162.8 (144-166)	NA	0.0640^1^
Proteinuria at M12 [g/24h; median (Q1-Q3)]	0.26 (0.10-0.34)	0.17 (0.12-0.25)	NA	0.5375^1^
HLA-A*02 recipients [n; (%)]	11 (64.7%)	4 (100%)	ND	0,2807^3^
Donor Specific Antibodies (DSA) [n; (%)]	1 (5.9%)	2 (50%)	NA	0.0727^3^
Post-Tx HCMV infection [n; (%)]	17 (100%)	/	NA	
HCMV infection time post-Tx [months; median (Q1-Q3)]	7.7 (3-11)	2.9 (1-2.5)	NA	**0.0160^1^**
Post-Tx HCMV reactivation [n; (% of HCMV^+^ recipients)]	4 (23.5%)	4 (100%)	NA	**0.0117^3^**
***HCMV anti-viral prophylaxis [n;(%)]***				
None	1(5.9%)	0	NA	0.0995^4^
Ganciclovir™	0	1(25%)	NA	0.0995^4^
Rovalcyte™	16(94.1%)	3(75%)	NA	0.0995^4^

NA, not applicable; ^1^Mann-Whitney U test, ^2^one-way ANOVA, ^3^Fisher’s exact test, ^4^ Khi2 test.

PBMC issued from KTR were prospectively isolated and stored in liquid nitrogen at the Centre de Ressources Biologiques (CRB, CHU Nantes, France). PBMC from HCMV^+^ HV were isolated by Ficoll density gradient (Eurobio, Les Ulis, France) and keep frozen until use. PBMC were thawed before use in RPMI-1640 medium (Gibco, Amarillo, Tx, USA) supplemented with 10% human serum (pool of male AB plasma, Sigma Aldrich, St-Louis, MI, USA), 2 mM L-glutamine (Gibco) and 100 U/mL penicillin (Gibco), 0.1 mg/mL streptomycin (Gibco). For KTR, blood samples were analysed before and after primary infection or reactivation. A single blood sample collected after a primary or a secondary infection was analyzed for all KTR and, for few recipients, 2-to-4 samples collected post-infection or reactivation were analyzed. A total of 20 blood samples from 17 KTR harvested after a HCMV primary infection and 12 samples from 4 HCMV+ recipients harvested post-reactivation were investigated for the detection of HCMV-specific CD8T cells and for phenotype analyses. A single sample harvested post-infection was analyzed for 35 HCMV+ HV. Samples from HCMV+ HV all correspond to chronic infection (viral latency). Patients, samples and individual time points toward HCMV infection/reactivation and transplantation are depicted in the **Supplemental Figure S1**. Samples from individual patients were all immunostained and acquired during the same experiment. For each time point, HLA-E_UL40_ and HLA-A2_pp65_ CD8T cells were concomitantly immunostained, quantified and phenotyped as described below.

### HCMV monitoring and *UL40* sequencing

The groups were defined according to the HCMV serology of the transplant recipients (HCMV^-^ or HCMV^+^) the day of transplantation and for HCMV^+^ hosts by the status of infection (primary, latent, reactivation): Symptomatic primary HCMV infection was defined as a mononucleosis-like syndrome with fever, malaise, lymphocytosis (>4.0 × 10^9^ lymphocytes/ml), and/or the presence of activated lymphocytes, combined with serology compatible with HCMV primary infection (positive HCMV IgM and/or IgG with low avidity). HCMV active infection (AI) was defined by having at least two consecutive PCR with a viral load (VL) > 3 log10, expressed as number of viral DNA copies (log10cop) per 10^6^ cells.

For HCMV monitoring, blood samples were collected for patient’s follow up or during the acute phase of HCMV infection. HCMV serology was performed using the LIAISON^®^ CMV IgG; 149 LIAISON^®^ CMV IgM and LIAISON^®^ CMV IgG Avidity tests (DiaSorin, Saluggia, Italia). Additional evidence of active HCMV replication was examined using HCMV-specific quantitative PCR on DNA extracted from whole blood, as we previously reported (21). The combination of positive HCMV IgM and/or IgG and positive PCR was used for confirmation of primary HCMV infection.

For UL40 sequencing, DNA from blood samples (1mL) was used as a template for the amplification HCMV UL40 DNA in a two-runs PCR as described (19, 22) using the following pairs of primers for a first long PCR: forward 5’-TCCTCCCTGGTACCCGATAACAG-3’ and reverse 5’-CGGGCCAGGACTTTTTAATGGCC-3’; and then for a second nested PCR: forward 5’-GGTAAGGGCACTCGTGAGGATGTGC-3’ and reverse 5’-TCCGAACGCTCGTGAGCAACAGTCG-3’. PCR products were purified using ExoSAP-IT^®^ USB (Affymetrix, Thermo Fisher Scientific). Bidirectional sequence was performed using the fluorescent BigDye terminator method (Big Dye version 1.1 Cycle Sequencing Kit, Applied Biosystems, Thermo Fisher Scientific) and sequence reactions were run on Applied Biosystems ABI Prism 3130 XL. Nucleotide and amino acid sequences were analysed using Seqscape software (version 2.5, Applied Biosystems). UL40 sequences were determined using of the UL40 sequences from HHV5 (Merlin strain), as references (NCBI Reference Sequence: NC_006273.2). The results of UL40 sequencing are reported in the supplemental **Table S1**.

### Production of HLA-E_UL40_ and HLA-A2_pp65_ tetramer complexes

Peptides from 4 HCMV UL40 protein variants (AA_15-23_: VMAPRTLIL, VMAPRTLLL, VMAPRSLLL and VMAPRTLVL) and the UL83 (pp65) protein (AA_495-503_: NLVPMVATV) were synthesized (purity>95%) and purchased from Genecust (Boynes, France). The HLA_peptide_ monomers HLA-E*01:01_UL40_ and HLA-A*02:01_pp65_ were produced by the recombinant protein core facilities (P2R, SFR Bonamy, Nantes Université, France) as we previously reported (19). HLA_peptide_ monomers were biotinylated, purified and tetramerized using APC- streptavidin (BD Biosciences, Le Pont de Claix, France) as we described previously (19).

### Immunostaining and multipanel flow cytometry

#### *Ex vivo* detection and quantification of HLA-E_UL40_ and HLA-A2_pp65_ CD8T cells

To detect anti-HCMV CD8T cells responses, PBMC (3x10^5^ per condition) were pre-incubated with a blocking anti-CD94 mAb (clone HP-3D9, PE, 5 µg/mL, BD Biosciences) for 25 min at 4°C to inhibit the binding of HLA-E-tetramers to CD94/NKG2A/C receptors. PBMC were then incubated with one of the different APC-labelled HLA-E_peptide_- or pHLA-A*02_pp65_-tetramers (10 µg/mL, 1 h, 4°C), before incubation (30 min, 4°C) with the following mAbs: anti-CD3 (clone SK7/Leu4, APC-H7, BD Biosciences), anti-CD8α (clone SK1, BV510, BD Biosciences) and anti- γδTCR (clone B1, BV421, BD Biosciences). In the present work, investigations of KTR’s samples were performed using either the exact UL40 peptide identified by the virus sequencing or using a peptide with the closest sequence (**Table S1**). For the analysis of HV, since the infecting strains were unckown, 3 of the most common UL40 peptides (VMAPRTLIL, VMAPRTLLL, VMAPRSLLL) were used for detection assays. A FMO condition (Fluorescence Minus One; all labelled-markers except one) without tetramer was performed for each sample as a negative control. Data acquisition was performed using BD LSR Fortessa™ X-20 and BD CANTO II™ and analyses were performed using BD DIVA™ Software v8.0 (BD Biosciences). Compensations were made by using anti-mouse κ chain Ab-coated beads (anti-mouse Ig, κ chain/negative control compensation particles set, BD Biosciences) incubated with corresponding mAb at the same concentration during 15 min at RT. Gating analysis strategy is depicted in (**Supplemental Figure S2**) and was identical for all samples. Briefly, lymphocytes were gated on the basis of their morphology in FSC-A/SSC-A, and doublets of cells were excluded using FSC-A/FSC-H and SSC-A/SSC-H dot plots. Frequency of tetramers^+^ CD8T cells subpopulations was determined after gating on the CD3^+^ TCR γδ^-^ cells.

#### Phenotype analysis of HCMV-specific CD8T cells

Phenotype was investigated for all the HCMV-specific CD8T responses identified in KTR and for all KTR included in the study. For HCMV+ HV, phenotype analysis was performed for 11 out of the 15 HV that display at least one HCMV-specific CD8T response, 4 HV with HCMV-specific CD8T response accounting for <0.5% were not included (**Table S2**). For phenotype analyses, immunostaining protocol included sequentially (1) an initial step of CD94 using blocking mAb, (2) an incubation step with HLA_peptide_ tetramers and (3) finally an incubation with anti-CD3, anti- γδTCR and anti-CD8α reported above as a preliminary steps. Next, PBMC were incubated in parallel with sets of mAbs for multipanel analyses as depicted in **Supplemental Figure S2**. Antibody included: BB700-anti-CD45RA (clone 5H9, BD Optibuild™, BD Biosciences), BV786-anti-CD45RO (clone UCHL1, BD Horizon™, BD Biosciences), PE-Cy7 anti-CCR7 (CD197, clone 3D12, BD Pharmingen™, BD Biosciences) BB515-anti-CD27 (clone M-T271, BD Horizon™), PE-anti-CD28 (clone CD28.2, BD Pharmingen™) and PE-anti-CD57 (clone NK1), BB700-anti-CD56 (clone B159), BB700-anti-HLA-DR (clone 46-6), BB515-anti-CD38 (clone HIT2), PE-anti-2B4 (CD244, clone 2-69), BV786-anti-4-1BB (CD137, clone 4B4-1), BB700-anti-PD-1 (CD279, clone EH12.1), PE-Cy7-anti-TIGIT (clone A15153G), BB515-anti-Tim-3 (CD366, clone 7D3), BB515-or FITC- anti-Lag-3 (CD223, clone T47-530), PE-Cy7-anti-KLRG1 (clone 2f1/KLRG1), BV786-anti-CX3CR1 (clone 2A9-1) and PE-anti-CD62L (clone DREG-56) all from BD Biosciences.

#### Phenotype analysis of CD56 expressing immune subsets

A 15-color mixed mAbs/pMHC tetramers panel was optimized for use on a Cytek Aurora (Cytek Biosciences, Fremont, CA, USA) spectral flow cytometry platform with a 5-laser configuration (laser excitation wavelenghts: 355nm, 405 nm, 488 nm, 561 nm, et 640 nm) as we previously described (23). Briefly, PBMC (1.10^6^/well) were costained using a multistep protocol. PBMC were incubated first with a viability marker (Fixable Viability Stain 440UV, BD Bioscience). Next, cells were incubated in successive steps with (1) BUV563-anti-NKG2C (clone 134591, BD Bioscience), (2) blocking anti-CD94 mAb (BD Bioscience), and (3) APC-labeled -HLA_peptide_ tetramers (HLA-E_UL40_ or HLA-A2_pp65_), Spark-NIR685-anti-CD19 (Clone HIB19, Biolegend), PerCepFluor710-anti-TCRγδ (clone B1.1, Thermo Fisher, Courtaboeuf, France) and APC-Fire750 anti-TCRγδ2 (clone B6, Biolegend), PE-anti-CD158 (clone HP-MA4, Biolegend, San Diego, CA, USA), BV785-anti-PD-1 (clone EH12.2H7, Biolegend), BV570-anti-CD4 (clone RPA-T4, Biolegend), FITC-anti-CD57 (clone HNK-1, Biolegend), BV510-anti-CD3 (clone OKT3, Biolegend), BUV395-anti-CD45RA (clone 5H9, BD Bioscience) BV805-anti-CD8 (clone SK1, BD Bioscience), BUV737-anti-CD56 (clone NCAM16.2, BD Bioscience) and BUV496-anti-CD16 (clone 3G8, BD Biosciences) mAbs. Cells were incubated with anti-NKG2C mAb before CD94 blockage to avoid interference due to CD94 blocking. The fluorescence intensities were then analyzed using SpectroFlo™ software version 2.2.0 (Cytek Biosciences). The frequency of major immune cell populations was determined using FlowJo™ Software v10 (BD Biosciences) based on manual gating strategies as we reported (23) and as shown in the **Supplemental Figure S3**. Identification of HLA-E_UL40_ CD8T cells based on selected phenotype markers or with tetramers was made using a gating strategy reported in **Supplemental Figure S3**.

#### Cell proliferation assays, cytotoxic capacity and cytokine production

Experiments were performed using PBMC from anonymous HCMV^+^ HV which possess either HLA-A2_pp65_ (n=4) or HLA-E_UL40_ (n=5) CD8T cells responses. For proliferation assays, PBMC were loaded with eF450-Cell proliferation dye (CPD, Invitrogen™, Carlsbad, CA, USA) and cultured for 4 days on 24-well culture plates either uncoated or coated with anti-CD3 (OKT3) and anti-CD8 mAbs (OKT8) in PBS (10µg/mL). At D4, PBMC were harvested and sequentially incubated first with CD94 blocking mAb, then with APC-labelled HLA-A2_pp65_ or HLA-E_UL40_ tetramers as above and finally immunostained with a cocktail of mAbs containing BV510-anti-CD3, PerCP eFluor710-anti-γδTCR, BUV805-anti-CD8 mAbs and Live-dead blue^®^ (Invitrogen™) to exclude dead cells. PBMC were washed twice in PBS before fluorescence analysis. The fluorescence intensities were measured with a 5-laser Cytek Aurora™ spectral flow cytometer (Cytek Biosciences) using SpectroFlo™ software version 2.2.0 (Cytek Biosciences). Cell division was quantified using Flowjo^®^ after gating on live CD3+ γδTCR- CD8+ tetramer positive (tet^+^) or negative (tet^-^) T cell populations as depicted on **Supplemental Figure S2**.

For the analysis of cytokine production and effectors of cytotoxicity, PBMC were incubated for 4h in the presence of phorbol 12-myristate 13-acetate (PMA) and ionomycine (Cell Stimulation Cocktail, eBioscience™) purchased from Sigma-Aldrich (St-Louis, MI, USA) with (for intracellular staining of cytokines and granzyme B) or without (for CD107a staining) brefeldin A and monensin in the presence or absence of HLApeptide tetramers. After treatment, PBMC were harvested and sequentially incubated first with CD94 bloking mAb, then with APC-labelled HLA-A2_pp65_ or HLA-E_UL40_ tetramers as above before immunostaining with cocktails of mAbs containing BV510-anti-CD3, PerCP 710-anti-γδTCR, BUV805-anti-CD8, Live-dead blue^®^ (Invitogen) and either BV605-anti-IL-2 (clone 5344.111, BD Biosciences), PerCPCy7-anti-GzmB (clone QA18A28, Biolegend), for panel 1, or PerCPCy7- anti-TNF (clone MAb11, Biolegend), BV605-IFNγ (clone B27, BD Biosciences) and PE-CD107a (clone H4A3, BD Biosciences), for panel 2. CD107a-specific mAb was added to the cells during the stimulation step. The expression of CD107a and GzmB were analyzed in parallel experiments but not on the same cells (no CD107a/GzmB costaining was performed). PBMC were washed twice in PBS before fluorescence analysis as reported above for proliferation and gating strategy was reported above and shown in **Supplemental Figure S2**.

### Cell culture, *in vitro* HCMV infection and mRNA analyses

We generated gene expression data from MRC-5 and primary human endothelial cells (EC) isolated from 5 individuals as we described (24); both cell types are commonly used in HCMV infection models. MRC-5 lung fibroblasts were purchased from the ATCC (CCL-171, Manassas, VA, USA) and grown in RPMI (Gibco) supplemented with 5% fetal calf serum (FCS, Gibco). Human primary vascular ECs were isolated by collagenase digestion as we previously reported (24) and used between passages 2 and 5. ECs were cultured in Endothelial Cell Basal Medium (ECBM) supplemented with 5% FCS, 0.004 mL/mL ECGS/Heparin, 0.1 ng/mL hEGF, 1 ng/mL hbFGF, 1 µg/mL hydrocortisone, 50 µg/mL gentamicin and 50 ng/mL amphotericin B (C-22010, PromoCell, Heidelberg, Germany). For HCMV production, an inoculate of HCMV isolate (VHL/E strain, kindly provided by Dr. Franck Halary, CR2TI INSERM UMR1064, Nantes, France) was used to produce a virus stock. For titration, a protocol adapted from (25) was used. For infection, cell monolayers at 50%-80% of confluence were infected with a HCMV (0.2 pfu/cell) for 4h in ECBM supplemented with 2% FCS to allow for virus adsorption. After incubation, culture medium was removed to eliminate free virus and cells are grown for the indicated period of time. Mock-infected cell samples were collected at each time, as mock RNAs can fluctuate over the HCMV infection time course. RNAs were isolated using TRIzol reagent (Invitrogen, Carlsbad, CA, USA), analyzed by Caliper LabChip GX Analyzer (Perkin Elmer Inc., Wellesley, MA, USA) for quantity and quality, and treated with DNase (Ambion, Austin, TX, USA) before reverse transcription (RT). Quantitative RT-PCRs (qRT-PCRs) were performed using the ABI PRISM 7700 sequence detection application program (PE Applied Biosystems, Foster City, CA, USA). For cellular mRNA quantification, means of C_t_ triplicates were normalized by the concomitant quantification of ribosomal protein lateral stalk subunit P0 (RPLP0, gene ID: 6175). Relative expression was calculated according to the 2^-ΔΔCt^ method, as previously described (26) and expressed as Fold-change ratios (HCMV/mock) after normalization to RPLPO. Cellular mRNAs were quantified by qRT-PCR with the following primers and probes purchased from Applied Biosystems: HLA-E (Hs03045171_m1), CD56 (Hs00941830_m1), CD112 (Hs01071562_m1), CD155 (Hs00197846_m1), RPLPO (Hs99999902_m1) Quantification of viral mRNAs in infected cells was achieved by qRT-PCR using SybrGreen^®^ technology with the following primers purchased from Eurofins Genomics (Ebersberg, Germany): UL11 (HHV5UL11for: 5’GCTGTTCAGGTACATTACC3’ and HHV5UL11rev: 5’GGACCGTATGACCAACGAAC3’ and UL40 (HHV5UL40for: 5’AAGGCGTAGTGATGATCGTTGGG3’ and HHV5UL40rev: 5’CCCGCCACGTTCGGTCTGGA3’), UL83 (HHV5UL83for: 5’AAGGCGGCCGCGTGTCATAACC3’ and HHV5UL83rev3: 5’GACGAAGAACTCGTAACCACCG3’) after validation as shown in the **Supplemental Figure S4**.

### Statistical analyses

Data are expressed as means ± SD, or as medians ± interquartile range between Q1 and Q3, or percentages. Appropriate statistical analyses were performed using GraphPad Prism^TM^ Software (GraphPad, San Diego, CA, USA) or R and the web application FaDa (https://github.com/danger-r/FaDAapp) when indicated. For comparisons of groups Fisher exact test, chi-square test, Mann–Whitney *U*-test, ANOVA, or 2-sided paired *t* test were used as appropriate. A p-value <0.05 was considered to represent a statistically significant difference.

## Results

### Generation and maintenance of long-lived HLA-E-restricted versus HLA-A2-restricted CD8T cell responses to HCMV

**Figure 1:**
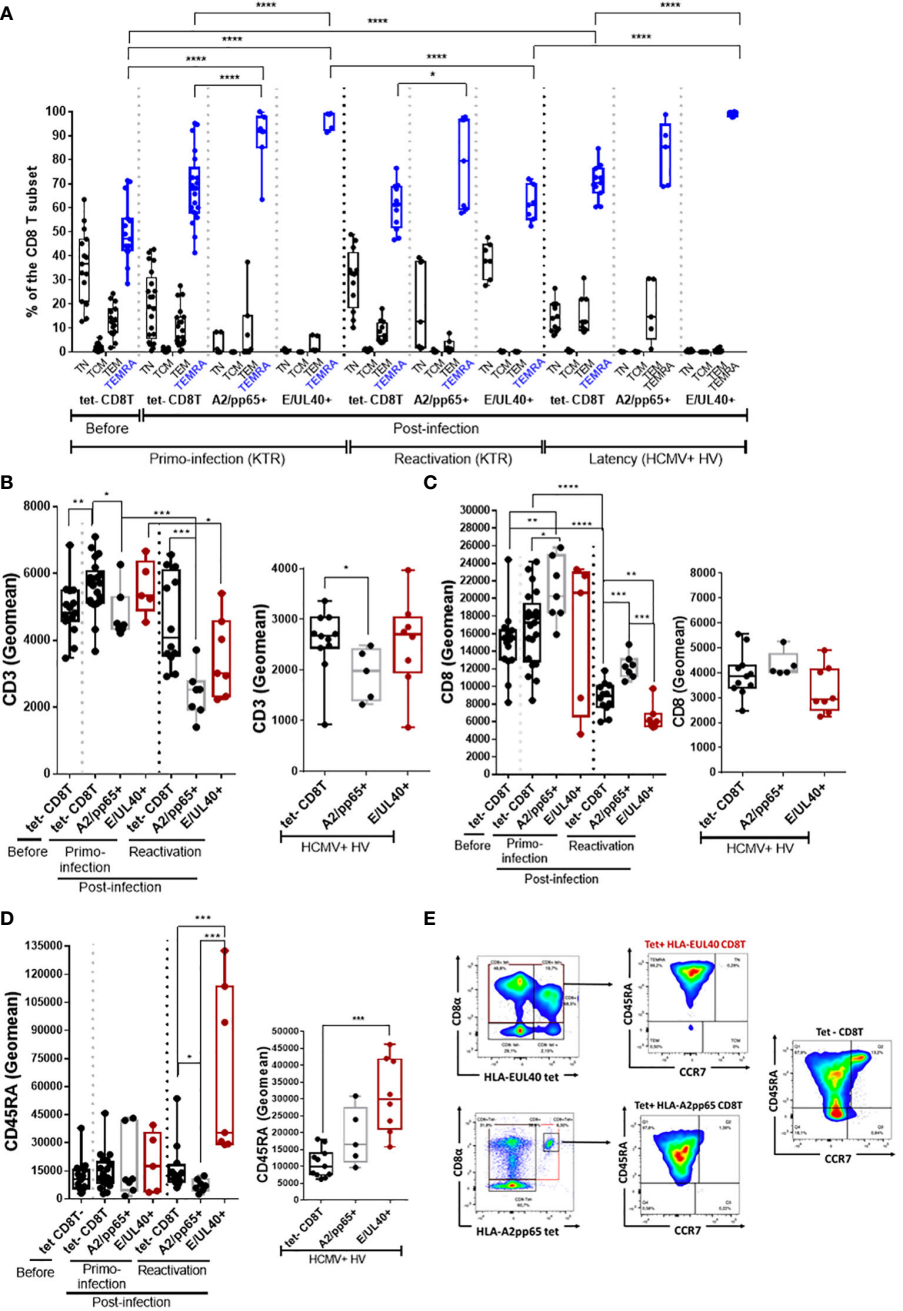
Detection and quantification of HCMV-specific CD8Tcell responses in KTR and HV and time course analysis of viral UL40 and UL83 (pp65) post-infection. **(A)** Donut charts showing the numberof hosts with HCMV-specific CD8Tcell responses (HLA-E_UL40,_ HLA-A2_pp65_, none) including KTR before and after a primary infection (R^-^), virus reactivation (R^+^) and in HCMV+ healthy controls (HV). The number of samples containing the differents HCMV-specific CD8T responses in the groups are indicated. **(B)** Box plots with median and interquartile values of the percentages of HCMV-specific CD8Tcell responses (HLA-E_UL40,_ HLA-A2_pp65_) detected in blood among total CD8Tcells in samples from KTR after a HCMV primary infection (7 A2pp65 responses and 5 EUL40 responses), a reactivation (7 A2pp65 responses and 7 EUL40 responses), and in HCMV^+^ HV (10 A2pp65 responses and 9 EUL40 responses). Each point corresponds to an individual CD8Tcell response. **(C–E)** Quantitative RT-PCR analysis of viral mRNA levels for UL40 and UL83/pp65 in HCMV-infected primary endothelial cells (n=5) **(C, D)**. Each point corresponds to an independant endothelial cell culture. **(E)** Quantitative RT-PCR analysis of viral mRNA levels for UL40 and UL83/pp65 in HCMV-infected MRC5 cells. Controls are uninfected cells. Results are medians from 5 independent endothelial cell cultures **(C, D)** or means ± SD from triplicate experiments for MRC5 cells **(E)** and are expressed as 2-ΔΔCt values normalized to the RPLP0 housekeeping gene. Statistical analysis was performed by Mann-Whitney *U*-test. P values: * for p < 0.05, ** for p<0.01 and *** for p<0.001.

To investigate the emergence and persistence of HCMV-specific CD8T cells we used PBMC samples collected from KTR before and after a primary infection post-transplantation. PBMC from 4 HCMV^+^ KTR who experienced an episode of HCMV reactivation, and with anti-HCMV CD8T cell responses previously detected, were also included as well as samples from HCMV^+^ HV to compare the frequency of CD8T cells responses after T cell priming, restimulation or during infection latency. Time points at sample collection before and after primary infection or reactivation are reported in **Supplemental Figure S1**. PBMC were analyzed by flow cytometry using HLA-A2_pp65_, HLA-E_UL40_ tetramers and a panel of mAbs to detect and quantify antigen-specific CD8T cells using a dedicated gating strategy shown in Fig.S2This allowed us to establish the frequency of hosts with the HLA-E_UL40_ and HLA-A2_pp65_ CD8 T and the frequency of these HCMV-specific CD8T cells populations among circulating CD8 T cells in the hosts. The panel of CD8T responses and the UL40 peptides recognized by the HLA-E_UL40_ CD8T cells in the patients and HV is reported in **Table S2**. No HCMV-specific CD8T cells were detected using our assays in samples collected before a primary infection. This findings confirm our previous study (19) indicating that HLA-E_UL40_ as well as HLA-A2_pp65_ CD8T cells appear in response to HCMV infection. First, the **Figure 1** shows that the percentage of individuals with HLA-E_UL40_ CD8T cells was similar for KTR with a primary infection and for HCMV^+^ HV with a chronic infection (24% and 26% of the total population, respectively). An equal frequency was also found for the detection of HLA-A2_pp65_ CD8T cells in both groups (29% of both KTR after a primary infection and of HCMV+ HV; **Figure 1A**). Next, the percentages of the respective HCMV-specific CD8T cell subsets among total blood CD8T cells was calculated to define the frequency of the responses (**Figure 1B**). The frequency for both types of anti-HCMV CD8T cells varies from 0.1% to >30% of total circulating CD8T cells according to hosts. These frequencies were similar in the different groups for HLA-E_UL40_ CD8T cells (mean values 1.1%, 2.5% and 4.1%, ns). In contrast, the frequency of HLA-A2_pp65_ CD8T cells was found significantly higher after reactivation (mean value: 1.4%) or at long term post-infection in HV (2.5%) than early after primo-infection (mean value: 0.3%) (**Figure 1B**) suggesting that development of HLA-E_UL40_ CD8T cells may occur earlier than those of HLA-A2_pp65_ CD8T cells. To investigate this discrepancy endothelial cells and fibroblasts were infected *in vitro* and viral mRNAs for UL40 and UL83 (pp65) were quantified by qRT-PCR (**Figures 1C–E**). Time courses analyses post-infection revealed that UL40 viral mRNA transcription occurs earlier and is quantitatively higher (around 100-fold) that UL83 mRNA supporting the idea that the emergence of HLA-E_UL40_ CD8T cells recognizing UL40 peptides may preceed the apparition of HLA-A2_pp65_ CD8T cells after infection.

### HLA-E_UL40_ CD8 TEMRA display distinctive low CD8 and high CD45RA expression compared to HLA-A2_pp65_ CD8 TEMRA

**Figure 2:**
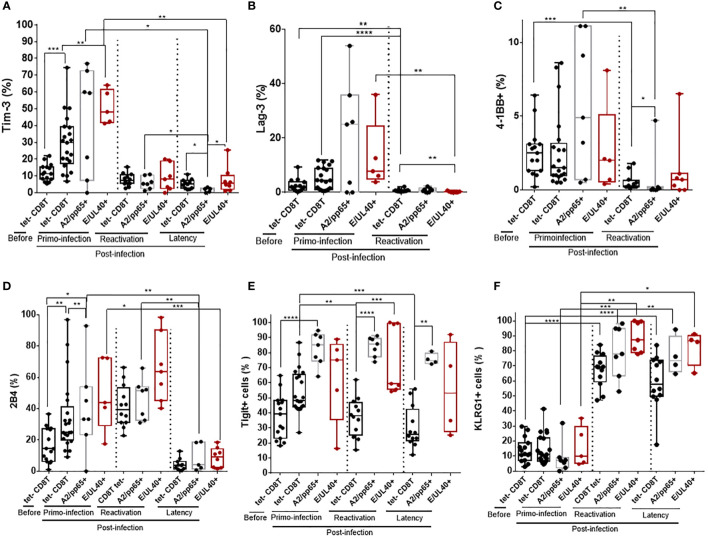
HLA-E_UL40_ memory CD8Tcells are TEMRA cells with distinctive levels for CD3, CD8 and CD45RA compared to HLA-A2_pp65_ CD8Tcells. After immunostaining and fluorescence acquisition CD8Tcell populations were defined using a dedicated gating strategy as reported in the Materials and methods section and in Fig S3 in order to identify tetramer^-^ CD8Tcells (tet-CD8T), HLA-E_UL40_ (E/UL40+) and HLA-A2_pp65_ (A2/pp65+) CD8 T cells. **(A–D)** Box plots representations of analyses performed on HLA-E_UL40_ CD8T cells from KTR with a primary infection (n=5), a reactivation (n=7), from HV at latency (n=9) and on HLA-A2_pp65_ CD8T cells from KTR with a primary infection (n=7), a reactivation (n=7), from HV at latency (n=10) and the respective tet- CD8T cells. Each point corresponds to a single CD8T response **(A)** Differentiation stage determined using CD45RA and CCR7 costaining to define naive T, central memory T cells (TCM), effector memory T cells (TEM) and terminally differentiated effector memory T cells reexpressing CD45RA (TEMRA). Data are expressed as percents of total CD8^+^ αβT cells in the respective subsets. Statistical analysis was performed by two-way Anova test. Comparative expression levels for CD3 **(B)**, CD8 **(C)** and CD45RA **(D)** in KTR before and post-infection (primary or reactivation) and HCMV^+^ HV (latency). Data are expressed as specific fluorescence intensities (geomeans). **(E)** Representative flow histograms illustrating CD8α and CD45RA expression at the surface of tetramer^-^ CD8Tcells (tet-CD8T), HLA-E_UL40_ (E/UL40+) and HLA-A2_pp65_ (A2/pp65+) CD8Tcells. **(B–D)** Statistical analysis was performed by Mann-Whitney *U*-test. p values: *for p < 0.05, **for p<0.01, ***for p<0.001 and ****for p<0.0001.

The phenotype of the various tetramer^+^ CD8T responses was compared by multipanel flow cytometry using the workflow and gating strategies depicted in **Supplemental Figure S3**. Firstly, the differentiation stage of anti-HCMV CD8T cells was investigated. After a primary HCMV infection or a reactivation, the homeostasis of overall circulating CD8T cells subsets changes toward a decrease in naive CD8T cells counterbalanced by an increase in effector CCR7^-^ memory T cells re-expressing CD45RA (TEMRA) cells (**Figure 2A**). Consistent with a rise in TEMRA cells in blood, we found that the vast majority of both HLA-E_UL40_ and HLA-A2_pp65_ CD8T cells populations display a phenotype specific of TEMRA after a primary infection, a reactivation and also during the chronic phase (at viral latency) of infection in HCMV^+^ healthy hosts. Compared to the pool of tetramer^-^ TEMRA CD8T cells in the various conditions, UL40- and pp65-specific memory T cells display significant changes in the expression of CD3, CD8 and CD45RA (**Figures 2B–E**). HLA-A2_pp65_ CD8 T cells express low CD3 and high CD8 while HLA-E_UL40_ CD8T display lower CD3 and CD8 post-reactivation and a higher CD45RA expression which persist long term post-infection as examplified in HV.

Considering the time course of infection, the expression of CD27 and CD28 was progressively lost after HCMV infection to reach a stable CD27^-^/CD28^-^, CCR7^-^, CD45RA^+^, CD8^+^ TEMRA phenotype during chronic infection such as in HV hosts with a latent HCMV infection (**Supplemental Figure S5**). After primoinfection there is a significant decrease in the coexpression of HLA-DR and CD38 for total and HCMV-specific CD8T cells and may reflect recent activation (**Supplemental Figure S5**). No specific changes were found during reactivation or latency.

### Common phenotype traits between HLA-E_UL40_ and HLA-A2_pp65_ CD8T cells and evolution according to the phase of HCMV infection

**Figure 3:**
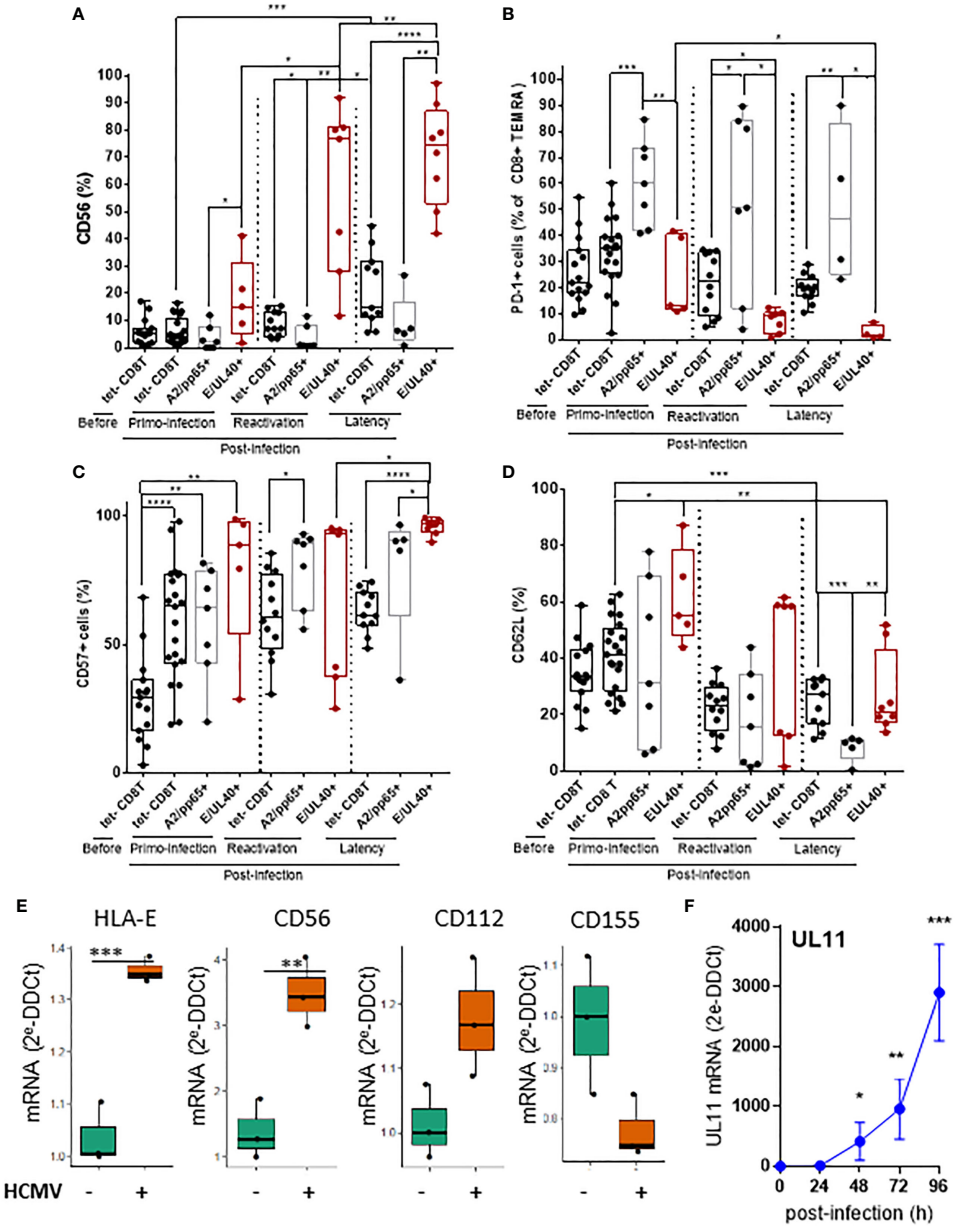
Immunophenotype of HLA-E_UL40_ and HLA-A2_pp65_ memory CD8 T cells after a primary HCMV infection, a reactivation in KTR and during chronic infection in HCMV+ HV. After immunostaining and fluorescence acquisition, CD8Tcell populations were defined using a dedicated gating strategy as reported in the Materials and methods section and in **Figure S3** in order to identify pMHC tetramer- CD8Tcells (tet-CD8T), HLA-E_UL40_ (E/UL40+) and HLA-A2_pp65_ (A2/pp65+) CD8Tcells. Box plots with median and interquartile values showing the specific expression for Tim-3 **(A)**, Lag-3 **(B)**, 4-1BB **(C)**, 2B4 **(D)**, Tigit **(E)** and KLRG1 **(F)**. Results are expressed as percents of positive cells in the different CD8 T cell subsets. **(A–F)** Box plots with median and interquartile values for analyses performed on HLA-E_UL40_ CD8T cells from KTR with a primary infection (n=5), a reactivation (n=7), from HV with chronic infection/latency (HV; n=9) and on HLA-A2_pp65_ CD8T cells from KTR with a primary infection (n=7), a reactivation (n=7), from HV with chronic infection/latency (n=10) and the respective tet^-^ CD8T cells. Each point corresponds to an individual CD8T response. Statistical analysis was performed by Mann-Whitney *U*-test. P values: *for p < 0.05, **for p<0.01, ***for p<0.001 and ****for p<0.0001.

Immunophenotyping was implemented to further characterize HLA-E_UL40_ CD8T cells responses and to compare them with conventional HLA-A2_pp65_ CD8T cells responses. Here we show that the phenotype of the two HCMV-specific CD8T populations evolves with the infection phase in KTR and HV hosts. Upon a primary infection, priming of HCMV-specific CD8T cells corresponds with a transient expression for Tim-3, Lag-3 and 4-1BB at the surface of tet+ CD8T cells (**Figures 3A–C**). The costimulatory molecule 4-1BB was expressed by a small subset of blood CD8 T cells (<10%) before and after primoinfection. The expression of 4-1BB was more disparate among hosts for HLA-E_UL40_ and HLA-A2_pp65_ CD8T cells after a primary infection but drop drastically upon HCMV reactivation. Tim-3 and Lag-3 were also not further expressed upon HCMV reactivation nor during latency in HCMV^+^ HV hosts for Tim-3. HCMV-specific CD8T priming and restimulation upon HCMV reactivation also associate with the expression of 2B4 while 2B4 was found to be expressed only by a minority of the tetramer^+^ CD8T populations in HV during latency (**Figure 3D**). Tigit was consistently expressed by HCMV-specific CD8T cells post-primoinfection, reactivation and during infection latency. After reactivation and long lived HLA-E_UL40_ and HLA-A2_pp65_ CD8T cells expressed consistently KLRG1, Tigit (**Figures 3E, F**). Thus Tim3, Lag-3, 4-1BB and 2B4 are hallmarks for T cell priming post-primary infection, and restimulaion for 2B4, while KLRG1 and Tigit are markers for restimulated and long lived HCMV-specific CD8T cells responses. These cell markers were equally expressed on HLA-E_UL40_ and HLA-A2_pp65_ CD8T cells.

### CD56 and PD-1 are cell markers discriminating HLA-E- from HLA-A2-restricted HCMV-specific CD8T cells

**Figure 4:**
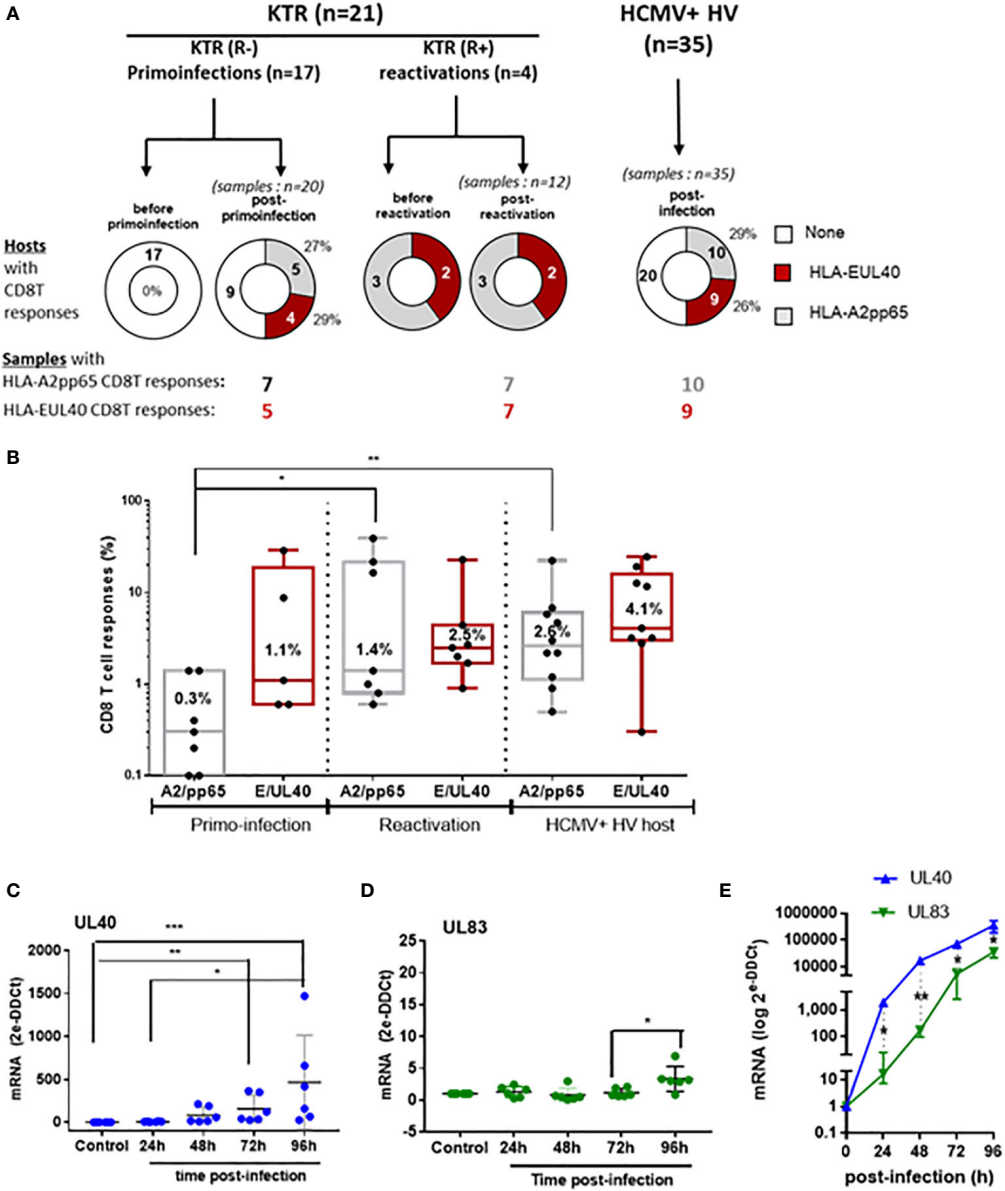
T cell markers discriminating HLA-E- from HLA-A2-restricted HCMV-specific CD8T cells and expression of related ligands on HCMV infected cells. After immunostaining and fluorescence acquisition, CD8Tcell populations were defined using a dedicated gating strategy as reported in the FigS3 in order to identify pMHC tetramer^-^ CD8Tcells (tet-CD8T), HLA-E_UL40_ (E/UL40+) and HLA-A2_pp65_ (A2/pp65+) CD8Tcells. **(A–D)** Box plots with median and interquartile values for analyses performed on HLA-E_UL40_ CD8T cells from KTR with a primary infection (n=5), a reactivation (n=7), HV (n=9) and on HLA-A2_pp65_ CD8T cells from KTR with a primary infection (n=7), a reactivation (n=7), HV (n=10) and the respective tet^-^ CD8T cells. Specific expression for CD56 **(A)**, PD-1 **(B)**, CD57 **(C)**, and CD62L **(D)** are shown. Results are expressed as percents of positive cells in the different CD8Tcell subsets. Each point corresponds to an individual CD8T response. Statistical analysis was performed by Mann-Whitney *U*-test. **(E, F)** Quantitative variations in mRNA levels for cellular **(E)** HLA-E, CD56, CD112, CD155 and viral **(F)** UL11 in primary human endothelial cell cultures after HCMV infection. Results from qRT-PCR are means ± SD obtained from 5 independent endothelial cell cultures and are expressed as 2-ΔΔCt values normalized to the RPLP0 housekeeping gene.R and the web application FaDa (https://github.com/danger-r/FaDAapp) were used for **(E)**. Statistical analysis was performed by Mann-Whitney U-test. P values: * for p < 0.05, ** for p<0.01, *** for p<0.005 and **** for p<0 .001.

Significant differences were found by comparing the phenotype of HLA-E_UL40_ and HLA-A2_pp65_ CD8T cells. HLA-E_UL40_ CD8T cells progressively acquire the expression of CD56 post-infection with up to 97% of HLA-E_UL40_ CD8T cells expressing CD56 during latency (**Figure 4A**). CD56 is selectively expressed on HLA-E_UL40_ CD8T cells detected after a primary infection. CD56 expression was found maximal post-reactivation and remains a feature HLA-E_UL40_ CD8T cells responses at latency, far away from infection. In contrast to HLA-E_UL40_, HLA-A2_pp65_ CD8T cells do not express CD56. Another marker discriminating HLA-E_UL40_ from HLA-A2_pp65_ CD8T cells is the inhibitory receptor PD-1. Post-infection, during acute phase as well as at latency, a majority of HLA-A2_pp65_ CD8 TEMRA express PD-1 (**Figure 4B**). In contrast, PD-1 was only expressed by a minority of HLA-E_UL40_ CD8 T (around 10%) after primary infection but was no longer expressed after reactivation and at latency. Differences in the expression for CD57 (**Figure 4C**) and CD62L (**Figure 4D**) were also found between the two CD8T cell responses. CD57 was found to be express on total (tetramer^-^) and by the two antigen-specific CD8T cells subsets after a primary infection and after HCMV reactivation. At latency, during the chronic phase of HCMV infection, CD57 is significantly higher on HLA-E_UL40_ CD8Tcompared to HLA-A2_pp65_ CD8T cells and total (tetramer^-^) CD8 T cells. Similarly at latency, CD62L was significantly higher on HLA-E_UL40_ CD8T cells compared to HLA-A2_pp65_ CD8T cells. Overall, HLA-E_UL40_ CD8 T cells are CD56+ CD57+ PD-1- CD8+ TEMRA cells whereas HLA-A2_pp65_ CD8T cells are CD56- CD57+ PD-1+ CD8+ TEMRA cells. In this study we used HCMV-infected cells to analyze whether the ligands of the receptors found expressed on HCMV-specific CD8T cells are expressed and either up- or down-regulated on infected cells. Our findings indicated that mirroring the phenotype traits of anti-HCMV CD8T cells, infected cells overexpress HLA-E (TCR ligand), CD56 (CD56 ligand) and the Tigit low affinity ligand CD112 but not CD155, the high affinity ligand for Tigit (**Figure 4E**) and thus may interact with/be target of HLA-E-restricted CD8T cells. Moerover, infected cells also express UL11, a potential ligand for CD45RA (**Figure 4F**).

### CD56^+^ PD-1^-^ HLA-E_UL40_ CD8T cells display rapid proliferation and effector functions

**Figure 5:**
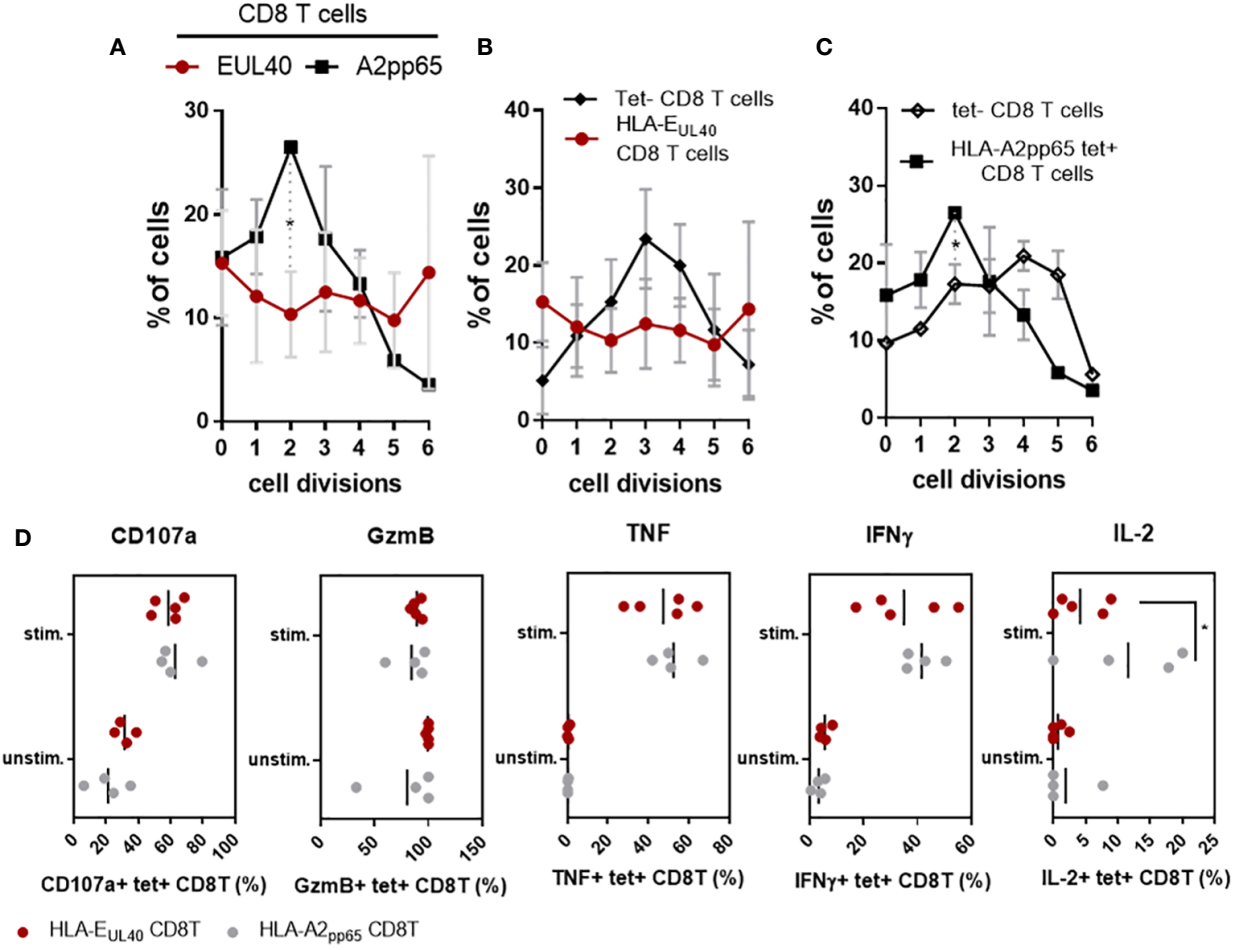
Proliferation and effector capacities for HLA-E_UL40_ versus HLA-A2_pp65_ CD8T cells. PBMC from HCMV^+^ HV possessing either HLA-A2_pp65_ (n=4) or HLA-E_UL40_ (n=5) CD8T cell responses were used. **(A–C)** For proliferation assays, PBMC were loaded with Cell Proliferation Dye and cultured for 4 days on culture plates either uncoated or coated with anti-CD3 and anti-CD8 mAbs before immunostaining. The number of cell division was quantified using Flowjo^®^ after gating on live CD3^+^ γδTCR^-^ CD8^+^ tetramer positive (tet+) HLA-E_UL40_ or HLA-A2_pp65_ and tetramer negative (tet-) T cell populations. Analyses comparing cell division for HLA-E_UL40_ and HLA-A2_pp65_ CD8T cell populations **(A)**, HLA-E_UL40_ and tet^-^ CD8T cell populations **(B)** and HLA-A2_pp65_ and tet^-^ CD8T cell populations **(C)** are shown. Data are expressed as percentages of cells in the cycles. **(D)** Level of membrane CD107a, granzyme B (GzmB) expression and intracellular TNF, IFNγ and IL-2 expressed by HLA-E_UL40_ and HLA-A2_pp65_ CD8T cell populations before (unstim) and after (stim) a 4h-stimulation assay in the presence of PMA/ionomycin. Each point represents an independant CD8T cell response. Data are expressed as percents of positive cells among tet^+^ CD8T cells. Statistical analysis was performed by Mann-Whitney *U*-test. P values: *for p < 0.05.

CD56 and CD57 are markers for cytotoxicity and proliferation, respectively (27) (28). Most of the pp65-specific and UL40-specific CD8T cells detected and analyzed in our study are TEMRA cells and do not express CD28 (Sup Fig S5). Consequently, in the set up experiments of our functional assays we tested the possibility to stimulate these CD8T cells *via* CD3 and CD8. Moreover, as shown in the **Figure 3**, pp65-specific and UL40-specific CD8T cells display distinctive levels of CD3 and CD8 expression. Thus our assay was designed to also investigate whether this difference of expression could affect their proliferation. Proliferation assays and cell division analyses indicated a faster proliferation for HLA-E_UL40_ CD8T cells compared to HLA-A2_pp65_ CD8T cells (**Figure 5A**). Moreover, proliferation for HLA-E_UL40_ CD8T cells was faster than the proliferation of total (tet^-^) CD8T cells (**Figure 5B**) while in contrary HLA-A2_pp65_ CD8T cells proliferation was slower than global CD8T cells (**Figure 5C**). Both HLA-E_UL40_ and HLA-A2_pp65_ CD8Tsubsets express Granzyme B and, after stimulation, CD107a indicating cytotoxic capacities. After stimulation HLA-E_UL40_ and HLA-A2_pp65_ CD8T cells similarly produce TNF and IFNγ while IL-2 production was higher for HLA-A2_pp65_ than for HLA-E_UL40_ CD8T cells (**Figure 5D**).

### CD56^+^ PD-1^-^ HLA-E_UL40_ CD8T cells display phenotype features shared with CD57^+^NKG2C^+^NK and δ2-γδT cells

**Figure 6:**
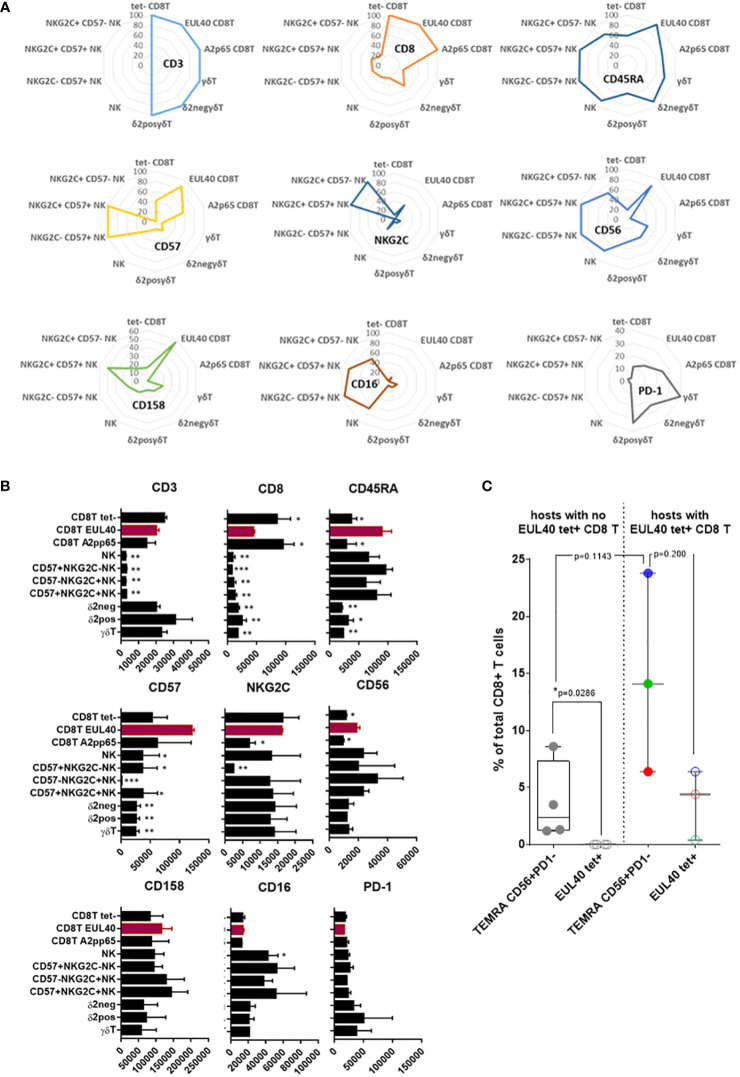
Phenotypic proximity of HLA-E_UL40_ CD8 αβT cells with NKG2C^+^CD57^+^ NK and δ2^-^γδT cells. Immunophenotypes comparing HLA-E_UL40_ CD8T cells to tetramer negative (tet-) and HLA-A2_pp65_ CD8 αβT, γδT and NK subsets based on their respective expression of CD3, CD8, CD45RA, CD56, CD57, PD-1, CD16, CD158 and NKG2C. Data were obtained from 4 individuals. **(A)** Radar plots showing the mean of percentages of expressing cells in the different cell subsets for each cell marker. **(B)** Histograms show the means ± SD of fluorecence intensity for expressing cells in the different cell subsets for each cell markers. P values for comparison between HLA-E_UL40_ CD8T and the other immune subsets: **(C)** Detection of CD8T cells using, in parallel experiments, a set of markers (CD3, CD8, CD45RA, CCR7, CD56, CD57, PD-1 and CD158) or HLA-E_UL40_ tetramers (EUL40 tet+) for cell gating on the same PBMC from HCMV^+^ individuals with (n=3) and without (n=4) HLA-E_UL40_ tet+ CD8T cells. Results are shown as box plots with medians and interquartiles values. Statistical analysis was performed by Mann-Whitney *U*-test. P values: *for p < 0.05, **for p<0.01, ***for p<0.005.

To further investigate the properties of CD56^+^ PD-1^-^ HLA-E_UL40_ CD8T cells we thought to achieved an analysis comparing the phenotype of HLA-E_UL40_ CD8T cells with other long lived immune subsets participating to the control of HCMV after infection. These subsets include total CD8αβT cells and the well described HLA-A2_pp65_ CD8T, total NK cells and subsets expressing CD57 and/or NKG2C, total γδT and δ2^-^γδT, δ2^+^γδT subsets from the same HCMV^+^ hosts. Immunophenotyping was performed by spectral flow cytometry and data are shown in the **Figure 6A, B**. This analysis indicates that while CD3 expression is a feature of both CD8^+^ αβT and γδT cells, CD8 and CD45RA are also expressed by γδT in particular δ2^-^γδT cells and by CD57^+^ NK subsets (**Figure 6A**). High and equal expression of CD45RA were found on HLA-E_UL40_ CD8T and NK cells. Among CD8T cells CD57 is selectively expressed by HLA-E_UL40_ and HLA-A2_pp65_ CD8T. In addition to these anti-HCMV CD8T subsets CD57 is restricted to CD57+NK cells. Interestingly, concerning the relative levels of expression, CD57 levels on HLA-E_UL40_ and HLA-E_pp65_ CD8T is significantly higher than the one on NK cells (**Figure 6B**). NKG2C was also only observed on HLA-E_UL40_ CD8T and on NKG2C+ NK cells. In contrast to CD57, CD56 was found common to HLA-E_UL40_ CD8T, γδT in particular δ2^-^γδT cells and by CD57^+^ NK subsets (**Figure 6A**) and expressed at similar level in these immune subsets (**Figure 6B**). Similarly CD158 is a another common feature of HLA-E_UL40_ CD8T, γδT in particular δ2^-^γδT cells and by CD57^+^ NK subsets. Finally, low or even no expression of PD-1 is a key feature shared by HLA-E_UL40_ CD8T, δ2^-^γδT cells and all NK cells. Therefore, CD8^low^, CD45RA^high^ CD56^+^, CD57^high,^CD158^+^, PD-1^-^ immune profile defines a set of HLA-E-restricted αβCD8T, γδT (mostly δ2^-^γδT) and CD57^+^ NK cells induced by HCMV infection. Finally, using either a set of these markers (CD3, CD8, CD45RA, CCR7, CD56, CD57, PD-1 and CD158) or HLA-E_UL40_ tetramers, for cell gating in parallel cytometry experiments, indicates that the phenotype CD3^+^ γδTCR ^-^ CD8^+^ CD45RA^+^ CCR7^-^ CD56^+^ CD57^+^ PD-1^-^ and CD158^+^ identifies a subset of CD8T cells present HCMV^+^ hosts independently of the detection of HLA-E_UL40_ tet^+^ CD8T (**Figure 6C**). Nevertheless, this CD8T subset is significantly enlarged in HCMV^+^ hosts with HLA-E_UL40_ CD8T detected with tetramers suggesting that this phenotype may includes HLA-E_UL40_ CD8T and allow their detection.

## Discussion

After viral infection, memory CD8T cells can provide efficient protection to re-infection due to their increased cytotoxic potential, cytokine secretion, and ability to respond to reinfection faster than naïve CD8T cells (4–6). Consistent with our previous findings (19), the present study investigating kidney transplanted patients with a primary HCMV infection post-transplantation shows that HLA-E-restricted CD8T cells, a subset of anti-HCMV CD8T cells, are frequently induced post-infection. We found that HLA-E-restricted CD8T are almost as frequent as the « immunodominant » conventional HLA-A2-restricted pp65-specific CD8T responses early after a primary infection as well as decades post-infection, during viral latency. Both UL40- and pp65-specific CD8T cells responses were detected for 25-30% of KTRs. A frequence similar to the one observed in HCMV^+^ chronically infected healthy individuals. These findings confirm the ability of KTR to develop robust conventional (HLA-Ia-restricted) and unconventional (HLA-E-restricted) HCMV-specific CD8 αβT cell responses owing immunosuppressive regimens. This also establish HLA-E-restricted CD8T cells as a major trigger of HCMV immunity. HLA-E-restricted UL40-specific CD8T cell responses appear early after HCMV primary infection and represent a stable pool of HCMV-specific CD8T cells representing 1-to-4% (median values), according to hosts, of total circulating CD8T cells that maintain for life. Early after primary infection the frequency of circulating HLA-E_UL40_ CD8T was found significantly higher than the frequency of HLA-A2_pp65_ T cells while these frequencies reach a similar rate after a secondary infection and in HCMV^+^ HVs (**Figure 1B**). This discripancy may suggest that HLA-E_UL40_ T cells appear earlier post-infection than HLA-A2_pp65_ T cell response. This could be explained by the fact that HCMV downregulates HLA class Ia expression but preserves HLA-E level by providing exogenous viral UL40 signal peptide to ensure the surface expression of HLA-E (13, 14, 29, 30). This phenomenom which may contribute to virus escape by promoting cellular inhibition through CD94/NKG2A triggering may also promote the rapid emergence of HLA-E-restricted CD8T cells while, in contrast, reduced expression for HLA-A2 by virus may delay pp65 antigen presentation and subsequent T cell priming. Our data further indicate that differential time course and steady state levels for viral mRNAs encoding UL40 and UL83 (pp65) in HCMV-infected cells may be additive factors which may also account for a delayed emergence of pp65-specific responses compared to UL40-specific responses. Future studies are needed to explore these hypotheses.

The principal objective of the present work was to decipher the phenotype of HLA-E_UL40_ in comparison to the HLA-A2_pp65_ CD8T cells. To this aim, we set up an experimental protocol for immunostaining using HLA-E_UL40 and_ HLA-A2_pp65_ tetramers and antibodies to cell surface molecules for multipanel flow cytometry analysis. Our results show that both UL40-sepcific and pp65-specific responses belong to TEMRA cells after a primary infection and display a stable CD27^-^/CD28^-^, CCR7^-^, CD45RA^+^, CD8^+^ TEMRA phenotype which persist lifelong in HCMV+ healthy hosts. However, in comparison to HLA-A2_pp65_ T cells, HLA-E_UL40_ T cells display distinctive levels of CD3 (CD3^high^), CD8 (CD8^low^) and CD45RA (CD45RA^high^) which may suggest specific activation capacity, affinity or/and regulation for HLA-E-restricted CD8T cells. CD45RA has been shown to be a receptor for the viral protein UL11 (31) and thus it could be speculated that elevated CD45RA may contribute to cellular contact with HLA-E-expressing cells *via* the binding to UL11 expressed on infected cells. Mechanistically, extracellular domain of the HCMV glycoprotein pUL11 has been shown to interact with CD45 in trans and to impair T cell signalling and functions (31) and promote IL-10 secretion (32). Here we found a rapid increase in the UL11 transcript level in HCMV-infected cells (**Figure 4F**) consistent with a potential role for the CD45/UL11 axis in the modulation of HLA-E_UL40_ CD8T cell activation and functions.

We found that CD3 and CD8 expression levels are other distinctive hallmarks for HLA-E_UL40_ CD8T cells. It is generally thought that the ability of CD8 coreceptor to enhance T cell responses is due to two main effects: first, binding of CD8 to MHC class I molecules helps to stabilize TCR-pMHC interactions and second, the recruitment of the Src kinase Lck to the TCR is more efficient upon CD8 binding to MHC molecules which enhance the initiation of TCR signaling (33). Consistent with our findings, it has been previously shown that T cell subsets are heterogeneous in terms of CD3 surface expression (34, 35). Among αβT cells differences have been established between CD4 and CD8T cells and for naive compared to memory T and regulatory T cells (34, 35). Moreover, γδT cells do express similar or even higher levels of CD3 than αβT cells (36) as observed in the present study (**Figure 6B**). Elevated level of CD3 on HLA-E_UL40_ CD8T cells should theoretically influence their capacity to respond to antigen since higher levels of membrane CD3 reached higher levels of activation (35).

**Figure 7:**
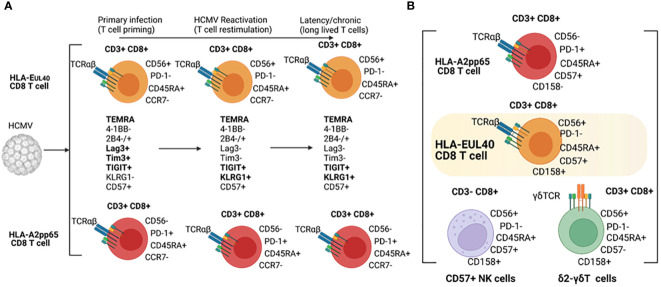
Phenotypical hallmarks of HLA-EUL40 CD8Tcells. **(A)** Phenotype of HLA-E_UL40_ CD8T in the course of HCMV infection and **(B)** Common phenotypic traits shared by HLA-E_UL40_ CD8T, CD8αβT, δ2^-^γδT and CD57^+^ NK cells.

Our results indicate that HLA-E_UL40_ and HLA-A2_pp65_ CD8 memory T cells share common phenotypic evolution post-infection with the coexpression of the regulatory receptors 2B4, Lag-3, Tim3 and 4-1BB as an early signature for the primary infection followed by the loss of Lag-3 and Tim3 and the gain of KLRG1 as a signature of T cell restimulation and long lived T cells during virus reactivation and chronic infection/latency, respectively. Immunophenotyping also revealed divergence between HLA-E_UL40_ and HLA-A2_pp65_ T cells as summarized in the **Figure 7**. A limitation to this study is that the comparison is limited to A2pp65 CD8T cells which represent a fraction of the repertoire of CD8T cells specific of HCMV and therefore does not capture the breadth of the conventional memory CD8T cells phenotypes against other HCMV antigens presented by HLA Ia molecules. Another limitation is the low number of patients especially in the reactivation group (n=4) thereby limiting the comparison between HCMV status at the time of kidney transplantation.

Distinctive membrane expression for CD56, CD57, CD62L and PD-1 are specific features of HLA-E_UL40_ T cells. HLA-E_UL40_ T cells express higher level of CD57 than do HLA-A2_pp65_ T cells. CD57 is a terminally sulphated carbohydrate determinant (glycoepitope) found on various surface glycoproteins, proteoglycans and glycolipids initially detected on subsets of NK cells. CD57 has been almost considered as a marker of replicative senescent T cells but CD57 also associates with a higher cytotoxic capacity, greater responsiveness to signaling, decreased responsiveness to cytokines (37)and failure to proliferate (38). HCMV drives expansion of NKG2C^ +^ NK cells which preferentially acquire CD57 (39) (40). In contrast to HLA-A2_pp65_ T cells, HLA-E_UL40_ T cells express no PD-1. PD-1 was found to restrict virus-specific CD8T cells responses during chronic infection by restraining proliferation during the effector phase (41). Previous studies have demonstrated that loss of PD-1 signals *in vitro* leads to increased phosphorylation of signaling molecules downstream of the TCR and costimulatory receptors (42). A feature of HLA-E_UL40_ CD8T cells is the lack of PD-1 expression. Considering the two cardinal features of exhausted CD8T cells which are the loss of effector capabilities and the sustained high expression of multiple inhibitory receptors including PD-1 (43) it could be argue that anti-HCMV HLA-E_UL40_ CD8T cells are not exhausted T cells.

Immunophenotyping of T and NK immune subsets allowed us to compare HLA-E_UL40_ CD8T cells to others CD8 αβT, γδT and NK subsets based on their respective expression of CD3, CD8, CD45RA, CD56, CD57, PD-1, CD16, CD158 and NKG2C. This study indicates that HLA-E_UL40_ CD8T cells share with NK cells a high level of CD45RA and CD56, the expression of CD57 (CD57^+^ NK cells) and NKG2C (NKG2C^+^ NK cells) for CD57^+^ and NKG2C^+^ NK subsets both induced by HCMV infection (39, 40). Consistent with a recent study (44), NKG2C expression is a feature that HLA-E_UL40_ CD8T share with NK cells. Since NKG2C binding to HLA-E_UL40_ mediates a strong activation of NK cells (45, 46), it can be speculated that NKG2C expression confer superior activity of the HLA-E_UL40_ CD8T compared to NK cells. Functional assays using cells expressing HLA-E pulsed with the UL40 peptides to detect potential synergistic or additive effect mediated by the engagement of TCR alone or together with NKG2C would be helpful to adress this point. Interestingly, γδT cells, in particular δ2^-^γδT, also express CD45RA, CD56, CD158 and CD8. Thus, these data further support the idea that HLA-E_UL40_ CD8T cells stay at the frontier between adaptive, memory NK, unconventional (γδT) and conventional (αβT) T cell responses and concur together with CD57^+^/NKG2C^+^ NK and δ2^-^γδT to the effector cell arsenal mobilized to cure infection (**Figure 7**). Functionally, expression of CD56 that define proximity for HLA-E_UL40_ CD8T cells with NK and γδT cells includes HLA-E_UL40_ CD8T cells in cytokine-induced killer cells (CIK). Indeed, CD56 expression is a key feature of CIK defined as an heterogeneous population of immune effector cells that feature a mixed T- and NK cell-like phenotype in their terminally-differentiated CD3^+^CD56^+^ subset (47, 48). They can be expanded *ex vivo* by culture of PBMC with anti-CD3 antibody and IL-2 [9]. CIK cells can recognize cancer cells which escape immune surveillance with low or even no MHC restriction, and CIK cells are currently evaluated in several randomized controlled trials as adjuvant cells for immunotherapies in cancer (48). Due to the low polymorphism of *HLA-E* gene and the relatively limited set of peptides presented by HLA-E molecules, HLA-E_UL40_ CD8T cells could also constitute a subset of cytotoxic memory T cells with low or even no HLA-restriction but with a high potential for adoptive cell therapy.

## Data availability statement

The raw data supporting the conclusions of this article will be made available by the authors, without undue reservation.

## Ethics statement

The studies involving human participants were reviewed and approved by CCPRB, CHU de Nantes, France. The patients/participants provided their written informed consent to participate in this study.

## Author contributions

Conceptualization, AR, NG, LD, BC. Methodology, AR, NG, LD, BC. Formal analysis, AR, NG, BC. Investigation, AR, NG, PG, BC, resources, MG, PG, CB-B, BC. Writing—original draft preparation, BC, AR. Supervision, BC. Funding acquisition, BC and CB-B. All authors contributed to the article and approved the submitted version.

## Funding

This research was funded by the “Association Grégory Lemarchal » and the « Association Vaincre la Mucoviscidose » (France), grant number RF20190502487 by l’Institut de Recherche en Santé Respiratoire des Pays de la Loire (France), grant number LSC 10280 and by the RHU project Kidney Transplantation Diagnostics Innovation (KTD Innov) funded by the ANR grant number 17-RHUS-0010. A.R. was recipent of a PhD grant from the Nantes Université (France).

## Acknowledgments

The authors thank the Recombinant Protein Facility (P^2^R, Structure Fédérative de Recherche “Francois Bonamy”, Nantes) for the production of pHLA-E_UL40_- and pHLA-A*02_pp65_-monomers. The authors also thank the Etablissement Français du Sang (EFS, Pays de la Loire, Nantes, France) and the Centre de Ressources Biologiques (CRB, CHU Nantes, France) for blood sample collection and harvesting. **Figure 7** has been created using Biorender.com.

## Conflict of interest

The authors declare that the research was conducted in the absence of any commercial or financial relationships that could be construed as a potential conflict of interest.

## Publisher’s note

All claims expressed in this article are solely those of the authors and do not necessarily represent those of their affiliated organizations, or those of the publisher, the editors and the reviewers. Any product that may be evaluated in this article, or claim that may be made by its manufacturer, is not guaranteed or endorsed by the publisher.
