# Supplementary material for: Distinctive phenotype for HLA-E- versus HLA-A2-restricted memory CD8 αβT cells in the course of HCMV infection discloses features shared with NKG2C+CD57+NK and δ2-γδT cell subsets
Source: Front Immunol. 2022 Dec 1;13:1063690. doi: 10.3389/fimmu.2022.1063690 (PMC9752567; doi:10.3389/fimmu.2022.1063690)
Supplement: Supplementary file 1 [file DataSheet_1.pdf]

Supplemental data

Figure S1

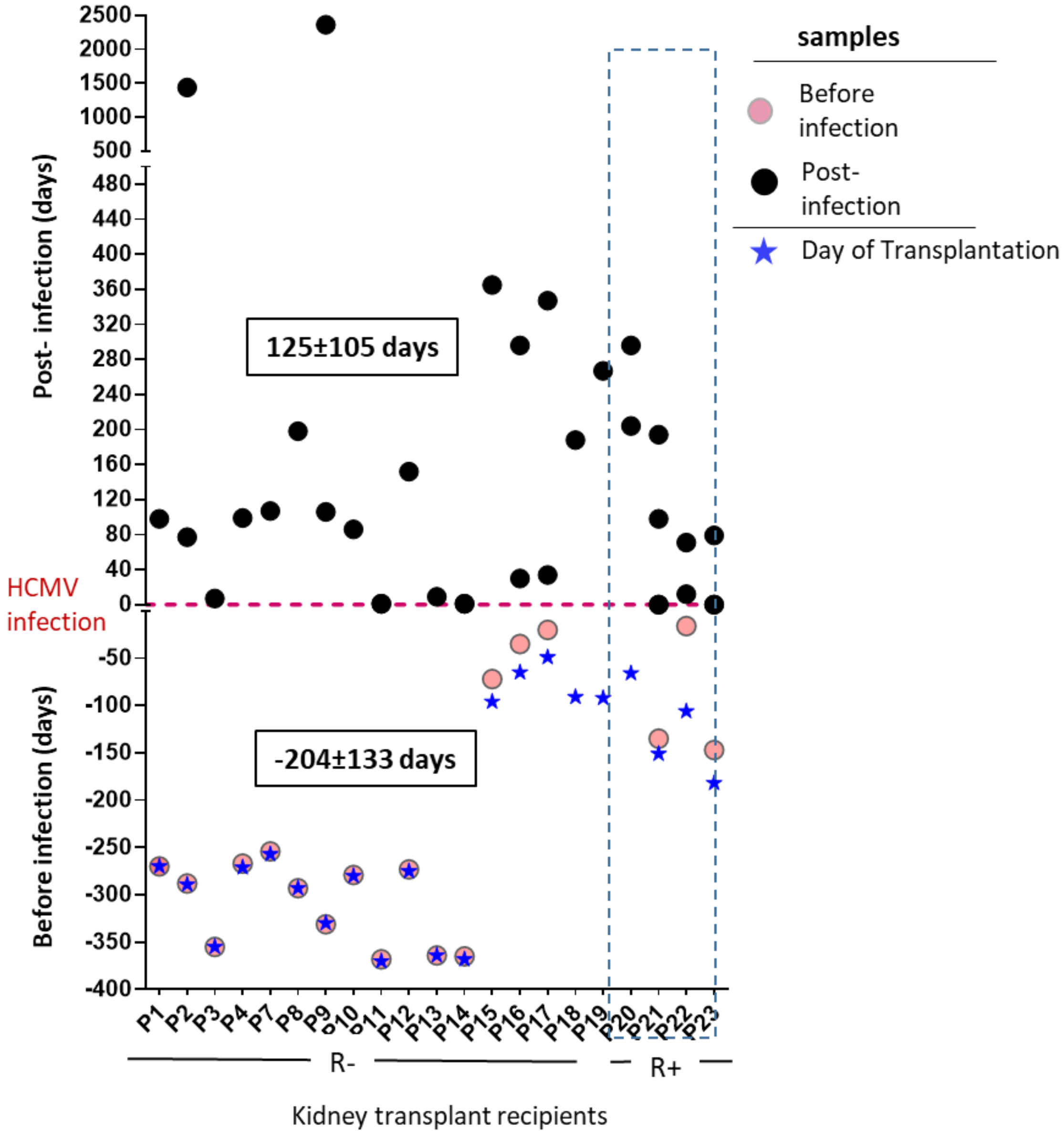

Figure S1. Time line of the blood samples harvested from kidney transplant recipients (n=21) before and after a primary infection (R-) or virus reactivation (R+) post-transplantation included in the study. The day of transplantation (star), the day of infection (D0, dashed red line) and the mean ± SD values of sampling time related to the day of infection (in frame) are indicated for all patients.

Figure S2

A

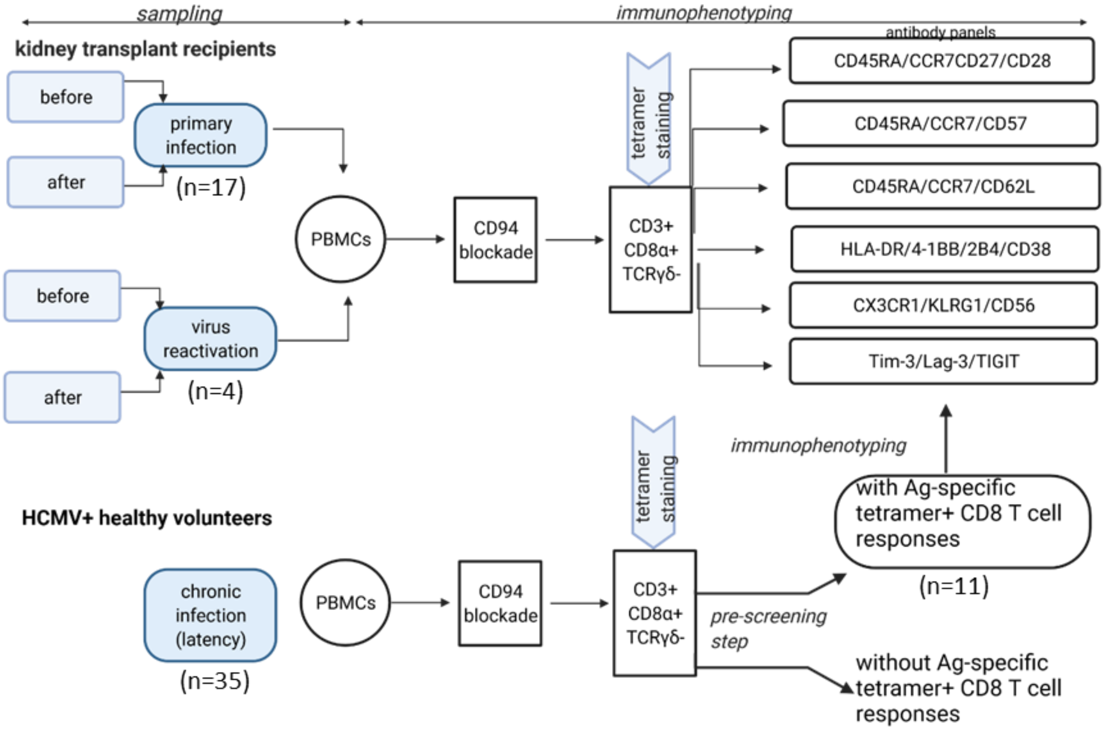

B

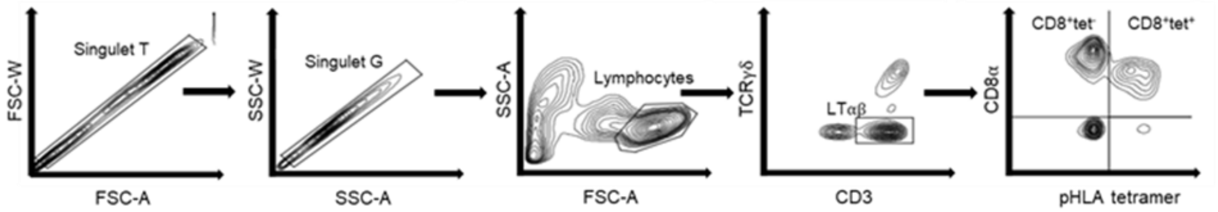

Figure S2. (**A**) **Workflow and cytometry panels** used for quantitative expression profiling of surface antigens on CD8T cell subsets from KTR and HV. (**B**) **A representative gating strategy** illustrating lymphocyte population subgated by the expression of CD3, γδTCR, CD8α and pHLA tetramer (tet). Cells were gated first on FSC-A *vs* FSC-H plots and then on SSC-H *vs* SSC-A plots to eliminate doublets. Lymphocytes were gated on an SSC-A vs. FSC-A dot plot. Lymphocytes were subgated using CD3 and γδTCR staining and then $CD3^+$ $γδTCR^-$ T cells were subgated using CD8α and pHLA tetramers. FSC-H: Forward scatter height. FSC-A: Forward scatter area. SSC: Side scatter. TCR : T cell receptor.

Figure S3

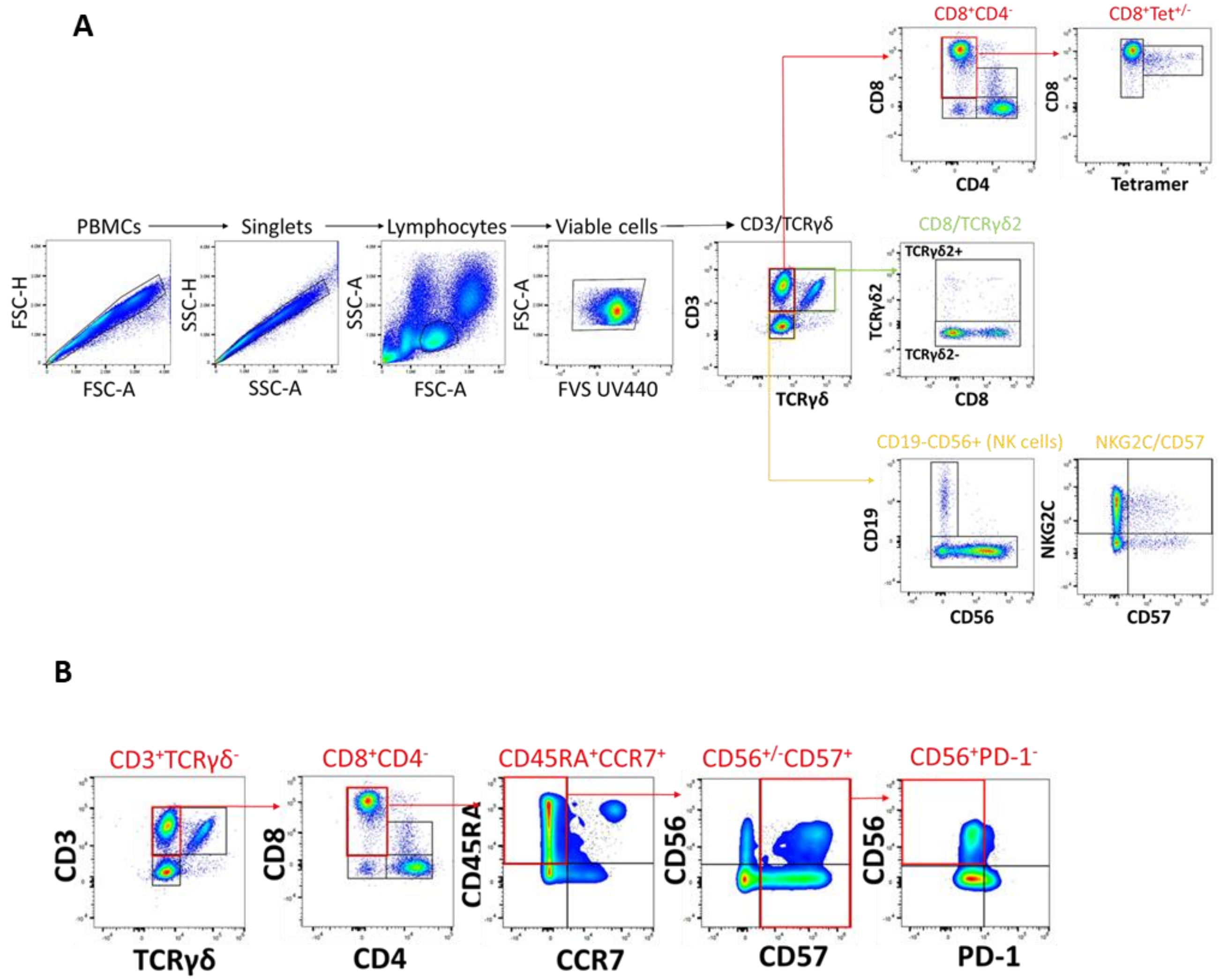

Figure S3. Representative gating strategies illustrating lymphocyte population gated by the expression of CD3, γδTCR and subsequent subgating (**A**) to identify the CD8 αβT populations (tet$^-$ and tet$^+$), γδT and NK cell subsets and (**B**) to identify the CD56$^+$ CD57$^+$ PD-1$^-$ TEMRA CD8 αβT populations.

Figure S4

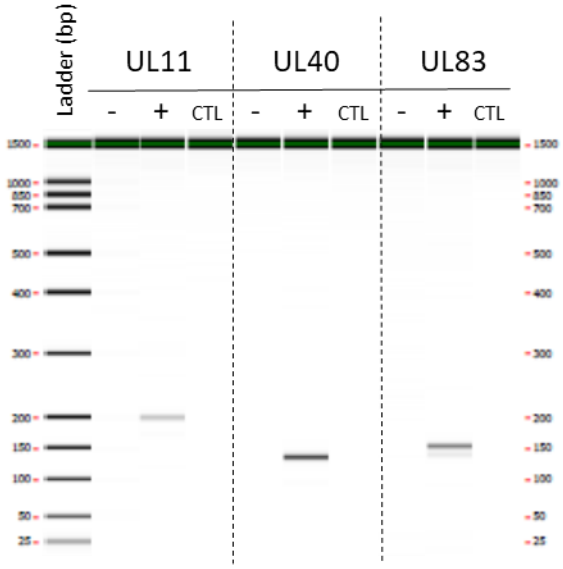

Figure S4. Representative RT-PCR amplicons obtained with UL11, UL40 and UL83 primer pairs using mRNA from MRC5 cells before (-) and after (+) HCMV infection. CTL indicates control PCR performed with no cDNA. Base pairs (bp).

Figure S5

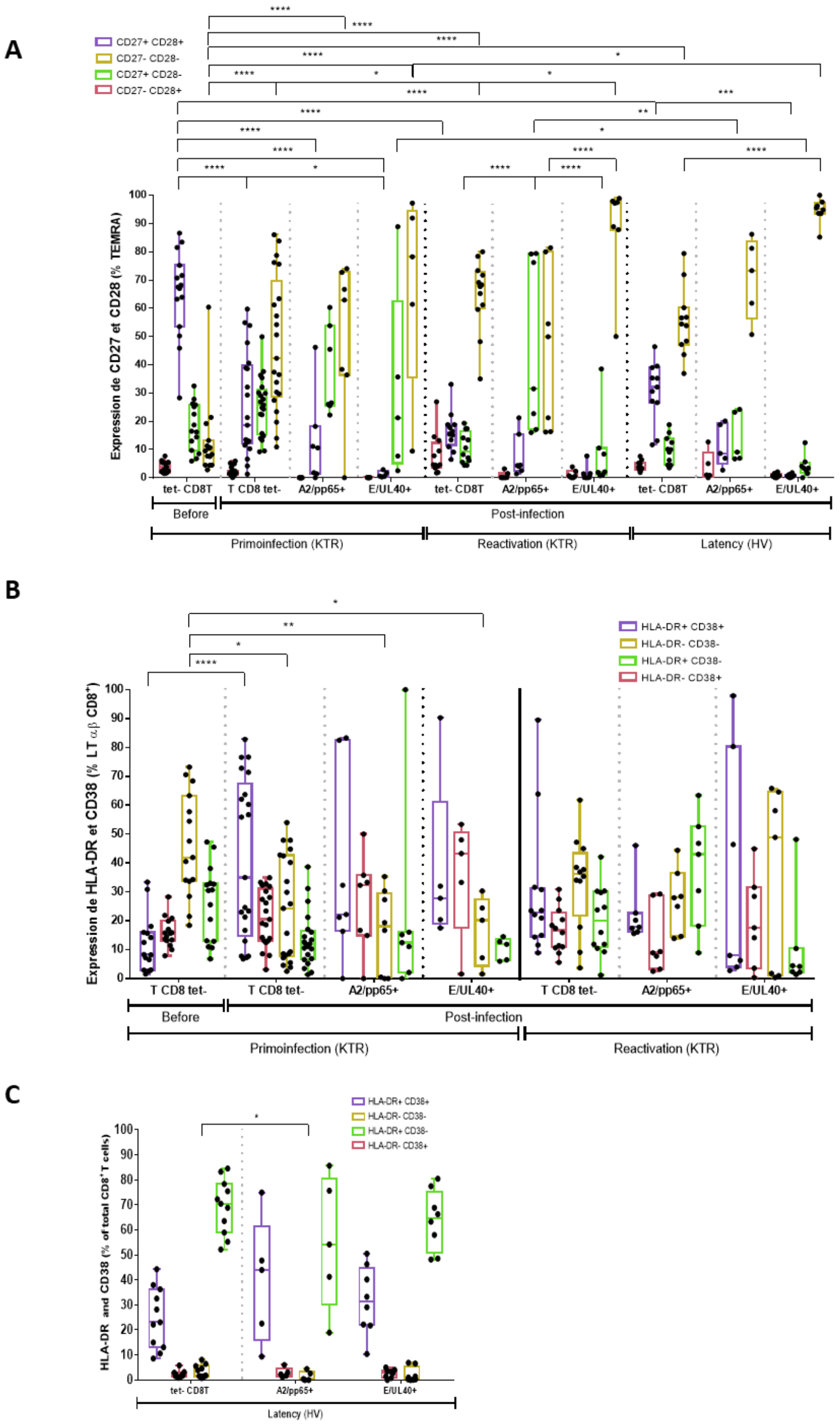

Figure S5. Coexpression of CD27/CD28 (**A**) and HLA-DR/CD38 (**B,C**) on the pHLA tetramer negative (tet-) and positive (A2/pp65+ and E/UL40+) CD8T susbets from KTRs and HCMV+ HV. Statistical analysis was performed by two-way ANOVA test. P values: * for $p < 0.05$, ** for $p<0.01$, *** for $p<0.001$ and **** for p<0 .0001.

Table S1. **Viral UL40 sequences identified in kidney transplant recipients and peptides loaded into HLA-E monomers for the detection of HLA-E$_{UL40}$ CD8T cells.**

	KTR	Viral UL40 (AA15-23) Sequences	Peptide used For detection
**HCMV primo infection**	P1	ND	VMAPRTLIL
	P2	VMGPRTLIL	VMAPRTLIL
	P3	VMAPRTLLL	VMAPRTLLL
	P4	VMAPRTLIL	VMAPRTLIL
	P7	LMAPRTLIL	VMAPRTLIL
	P8	VMAPRSLLL	VMAPRSLLL
	P9	ND	VMAPRTLIL
	P10	VMTPRTLIL	VMAPRTLIL
	P11	VVAPRTLLL	VMAPRTLLL
	P12	VIAPRTLIL	VMAPRTLIL
	P13	VMASRTLFL	VMAPRTLIL
	P14	ND	VMAPRTLIL
	P15	VMA(-----)L	VMAPRTLIL
	P16	ND	VMAPRTLIL
	P17	VMAPRTLLL	VMAPRTLLL
	P18	VMAPRTLLL	VMAPRTLLL
	P19	VMAPRTLAL	VMAPRTLVL
**HCMV reactivation**	P20	ND	VMAPRTLIL
	P21	VMAPRTL(I/L)L	VMAPRTLLL
	P22	VMAPRSLLL	VMAPRSLLL
	P23	VMAPRT(LL/VL)L	VMAPRTLLL

ND : not determined.

Table S2. Hosts, samples and peptide-specific CD8T cell responses detected and analyzed.

	Hosts	Samples post-infection (n)	HLA-A2 /pp65	HLA-E /UL40
**KTR primary infection**	P8	1	0	**VMAPRSLLL**
	P9	2	**pp65**	0
	P11	1	**pp65**	0
	P14	1	0	**VMAPRTLIL**
	P16	2	**pp65**	0
	P17	2	**pp65**	**VMAPRSLLL**
	P18	1	**pp65**	ND
	P19	1	0	**VMAPRTLVL**
**KTR reactivation**	P20	2	**pp65**	ND
	P21	3	ND	**VMAPRTLLL**
	P22	4	**pp65**	**VMAPRSLLL**
	P23	3	**pp65**	ND
**HCMV+ HV**	***HV4***	1	pp65	**VMAPRTLLL**
	HV5	1	pp65	0
	HV6	1	0	VMAPRTLIL
	HV9	1	**pp65**	**VMAPRTLIL**
	HV14	1	**pp65**	**VMAPRTLLL**
	HV15	1	**pp65**	0
	HV18	1	pp65	0
	HV19	1	0	**VMAPRTLIL**
	HV20	1	0	**VMAPRTLIL**
	HV21	1	0	**VMAPRTLLL**
	HV24	1	pp65	0
	HV25	1	pp65	**VMAPRTLLL**
	HV27	1	0	**VMAPRTLIL**
	HV30	1	**pp65**	0
	HV35	1	**pp65**	0

ND: not determined ; CD8T cell responses indicated in bold were further investigated for phenotype analyses.
